# Anti-toxoplasma activity and DNA-binding of copper(II) and zinc(II) coordination compounds with 5-nitroimidazole-based ligands

**DOI:** 10.1007/s00775-023-02029-7

**Published:** 2023-12-15

**Authors:** Rubí Navarro-Peñaloza, Jhony Anacleto-Santos, Norma Rivera-Fernández, Francisco Sánchez-Bartez, Isabel Gracia-Mora, Ana B. Caballero, Patrick Gamez, Norah Barba-Behrens

**Affiliations:** 1 Departamento de Química Inorgánica, Facultad de Química, Universidad Nacional Autónoma de México, Ciudad Universitaria, Coyoacán, 04510 Mexico City, Mexico; 2 Departamento de Microbiología y Parasitología, Facultad de Medicina, Universidad Nacional Autónoma de México, Ciudad Universitaria, Coyoacán, 04510 Mexico City, Mexico; 3 Unidad de Investigación Preclínica (UNIPREC), Facultad de Química, Universidad Nacional Autónoma de México, Ciudad Universitaria, Coyoacán, 04510 Mexico City, Mexico; 4 nanoBIC, Departament de Química Inorgànica i Orgànica, Secció Química Inorgànica,, Universitat de Barcelona, Martí i Franquès 1–11, 08028 Barcelona, Spain; 5 Institute of Nanoscience and Nanotechnology (IN2UB), Universitat de Barcelona, 08028 Barcelona, Spain; 6 Catalan Institution for Research and Advanced Studies (ICREA), Passeig Lluís Companys 23, 08010 Barcelona, Spain

**Keywords:** Coordination compounds, Nitroimidazole ligands, Anti-toxoplasma activity, Copper(II) and zinc(II), DNA-damage

## Abstract

Tetrahedral copper(II) and zinc(II) coordination compounds from 5-nitroimidazole derivatives, viz*.* 1-(2-chloroethyl)-2-methyl-5-nitroimidazole (cenz) and ornidazole 1-(3-chloro-2-hydroxypropyl)-2-methyl-5-nitroimidazole (onz), were synthesized and spectroscopically characterized. Their molecular structures were determined by X-ray diffraction studies. The complexes [Cu(onz)_2_X_2_], [Zn(onz)_2_X_2_], [Cu(cenz)_2_X_2_] and [Zn(cenz)_2_X_2_] (X^−^ = Cl, Br), are stable in solution and exhibit positive LogD_7.4_ values that are in the range for molecules capable of crossing the cell membrane via passive difussion. Their biological activity against *Toxoplasma gondi* was investigated, and IC_50_ and lethal dose (LD_50_) values were determined. The ornidazole copper(II) compounds showed very good antiparasitic activity in its tachyzoite morphology. The interaction of the coordination compounds with DNA was examined by circular dichroism, fluorescence (using intercalating ethidium bromide and minor groove binding Hoechst 33258) and UV–Vis spectroscopy. The copper(II) compounds interact with the minor groove of the biomolecule, whereas weaker electrostatic interactions take place with the zinc(II) compounds. The spectroscopic data achieved for the two series of complexes (namely with copper(II) and zinc(II) as metal center) agree with the respective DNA-damage features observed by gel electrophoresis.

**Graphical abstract:**

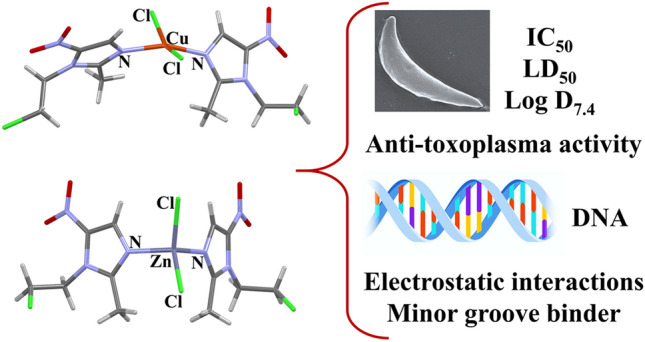

**Supplementary Information:**

The online version contains supplementary material available at 10.1007/s00775-023-02029-7.

## Introduction

5-Nitroimidazole derivatives are a group of heterocyclic compounds of broad spectrum against Gram-positive and Gram-negative bacteria, protozoa, and helminth that cause infection on the human body [1–3]. Metronidazole (mtz) was the first derivative to be used due to its potent activity against the parasite *Trichomonas vaginalis*, which causes trichonomia disease. It is currently used against amebiasis caused by *Etamoeba histolytica* and *Giardia lambia*, and for stomach ulcers caused by *Helicobacter pyroli* [1–4]. After the discovery of metronidazole, a second and third generation of 5-nitroimidazole drugs with different chains at the N1-position of the imidazole were developed, such as tinidazole (tnz), secnidazole (snz) and ornidazole (onz) [1, 2].

Regarding the mechanism of action, 5-nitroimidazoles are considered as pro-drugs which require intracellular bioactivation through three essential steps: (1) entry of the compound into the cell by passive diffusion, (2) enzymatic reduction (type I or type II) of the nitro group and (3) damage by rupture of DNA strands and subsequent cell death [1–7]. The type I mechanism is oxygen-insensitive and consists in sequential two-electron reductions of the nitro group generating relatively stable nitroso and hydroxylamine intermediates. On the other hand, the type II mechanism (one-electron reduction) is oxygen sensitive and gives rise to a nitro radical anion R-NO_2_^⋅−^ which reacts with molecular oxygen to form a superoxide anion and subsequent reoxidation to a nitro group [7]. This process is responsible for the toxic effects of the nitro groups that cause gastrointestinal diseases, carcinogenicity, hepatotoxicity and mutagenicity. Resistance of microorganisms to these drugs have also been observed [6–10].

Due to reported clinical resistance and side effects, the use of 5-nitroimidazoles as ligands to generate coordination compounds has been reported as an alternative for the treatment against parasites and bacteria [11]. We have been interested in investigating the biological activity of a series of coordination compounds with nitroimidazole derivatives; among them tinidazole complexes were synthesized. The tetrahedral coordination compounds [Cu(tnz)_2_Br_2_] and [Zn(tnz)_2_Br_2_] were tested against the helminth *Euryhaliotrema perezponcei,* which affects flamingo snapper fishes that are consumed by humans. The in vitro and in vivo assays carried out presented a parasite mortality percentage of 100% for [Cu(tnz)_2_Br_2_] [12, 13]. Characterization by UV–Vis spectroscopy, fluorescence spectroscopy and gel electrophoresis showed that these compounds are capable of causing DNA-damage through binding to its minor groove via electrostatic interactions and by oxidative cleavage [14]. Recent theoretical studies of the DNA recognition process with Molecular Dynamics and the Quantum Theory of Atoms in Molecules, provided results that were consistent with the experimental findings [15].

Previously, the tetrahedral mononuclear compounds [Cu(onz)_2_Cl_2_] and [Zn(onz)_2_Cl_2_] have been reported with the onz ligand. The zinc(II) complex showed higher activity against amoeba *Entamoeba histolytica* than the free ligand (MIC = 6.25 µM and 12.5 µM, respectively) [16, 17].

Toxoplasmosis is an infectious zoonotic disease caused by the apicomplex opportunistic parasite *Toxoplasma gondii*, which infects humans and all homoeothermic vertebrates. It is one of the most common infections in humans; it is estimated that approximately one third of the world's population is infected with latent toxoplasmosis [18, 19]. The disease can go unnoticed clinically in immunocompetent patients, but it causes fatal complications in immunocompromised patients, such as those living with HIV, who commonly have brain lesions due to this parasite [18, 20, 21]. Women of reproductive age and fetuses are also affected groups [18–22]. *T. gondii* can be transmitted by (i) oral ingestion of food and water contaminated by parasite oocysts that are shed in cat feces, (ii) consumption of raw or undercooked meat containing tissue cysts, and (iii) congenital transmission from mother to fetus [21]. The parasite is heteroxenous, completing its evolutionary cycle in two or more hosts, during this cycle it fulfills three different morphologies, namely: oocysts, which is the phase in which it can survive for long periods of time outside the host; bradyzoites or cysts, which is the slow replicating form and has a great affinity for neuronal and muscular tissue; and the tachyzoite, which is the rapidly replicating form [19–23]. Currently, pyrimethamine-sulfadiazine is the combination of choice to treat acute toxoplasmosis, with an effectiveness of 75–89%. However, resistance to both drugs has been reported and they are known to cause serious host side effects including agranulocytosis, hypersensitivity reactions, leukopenia, neutropenia, skin and liver necrosis, Stevens-Johnson syndrome [19, 24].

Due to the prevalence of toxoplasmosis and the complications that occur in immunocompromised patients, various studies have been focused on finding safe drugs with new mechanisms of action that are effective and non-toxic to patients [25]. An alternative anti-toxoplasma treatment is provided by natural products, like for instance those extracted from *Pleopeltis crassinervata* [26]. Some, pyridine-based coordination compounds of iron(III) cobalt(II) copper(II) and zinc(II) were also described as active agents against *T. gondii* [27–31]. The most effective coordination compounds, which were recently patented, are the dinuclear copper(II) complexes [(HL1)Cu(µ-Cl)_2_Cu(HL1)]Cl_2_∙H_2_O and [(H_2_L2)Cu(µ-Cl)_2_Cu(H_2_L2)]Cl_2_∙6H_2_O, with IC_50_ values of 0.78 and 3.57 µM, respectively [30, 31].

**Fig. 1:**
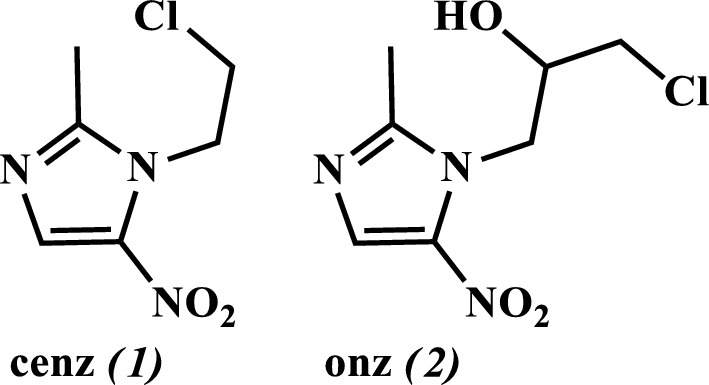
Representation of the structures of the 5-nitroimidazole-based ligands cenz and onz

Given the prevalence of toxoplasmosis throughout the world, the associated complications in immunocompromised patients, the low efficacy of currently available drugs, and the relevance of 5-nitroimidazole derivatives for the treatment of parasitic diseases, we have been interested in the development of new coordination compounds of copper(II) and zinc(II) with 1-(2-chloroethyl)-2-methyl-5-nitroimidazole (cenz) and 1-(3-chloro-2-hydroxypropyl)-2-methyl-5-nitroimidazole (onz), Fig. 1. Herein, their structural and physicochemical properties, interactions with DNA, as well as their anti-toxoplasma activity will be discussed.

## Experimental

### Materials and reagents

The reagents and solvents were used without further purification. The ligand 1-(3-chloro-2-hydroxypropyl)-2-methyl-5-nitroimidazole (onz) was obtained from Sigma Aldrich, as well as thionyl chloride, sulfadiazine, Hoechst 33258, cacodylic acid, (3-(*N*-morpholino) propanesulfonic acid) (MOPS), ascorbic acid, hydrogen peroxide, SYTOX^®^ Green, SYBR™ Safe and the calf thymus DNA. The metal salts: Na_2_CO_3_, Na_2_SO_4_, CuCl_2_⋅2H_2_O, CuBr_2_, ZnCl_2_ and ZnBr_2_, and solvents: acetonitrile, ethyl acetate, hexane, ethanol, dimethyl sulfoxide, and 1-octanol were obtained from J.T. Baker. NaCl was purchased from Fisher, TBE 10X from Invitrogen, sodium salt of calf thymus DNA (ct-DNA, type I fibrous) from Sigma-Aldrich, plasmid pBR322 (4361 bp, 0.25 mg mL^−1^) from Thermo Scientific, agarose from Ecogen and ethidium bromide 10 mg mL^−1^ from Promega.

### Synthesis of 1-(2-chloroethyl)-2-methyl-5-nitro-1H-imidazole (cenz) (*1*)

The synthesis of cenz was previously reported [32, 33]. However, light modification of the reaction conditions were used, and the ligand was prepared as follows. 5 mL (70 mmol) of thionyl chloride were slowly added, under anhydrous conditions and inert atmosphere, to two grams (12 mmol) of metronidazole in an ice bath. The reaction was kept under stirring and heated with a sand bath for 4 h at 65 °C. Thereafter, a Na_2_CO_3_ solution was added slowly, in an ice bath until reaching pH 9. Subsequently, extractions were carried out with ethyl acetate (3 × 15 mL); from the combined organic phase was evaporated under reduced pressure and a beige product was obtained. Yield: 89%, ^1^H NMR (400 MHz, d^6^-DMSO): δ/ppm = 8.02 (s, 1H, Iz-H); 4.63 (t, 2H, CH_2_); 3.97 (t, 2H, CH_2_); 2.46 (s, 3H, CH_3_). ^13^C{^1^H} NMR (400 MHz, d^6^-DMSO): δ/ppm = 152.28; 138.93; 133.69; 47.36; 43.64; 14.77 (Fig. S1). FT-IR (ATR, cm^−1^) 3017 ν(CH_3_), 2930 ν(CH_2_), 1520 ν(C=N), 1458 ν_as_(NO_2_), 1422 ν(N–C–N), 1366 ν_s_(NO_2_), 1259 ν(C–N), 1190 ν(N–O). Anal. Calc. for C_6_H_8_N_3_O_2_Cl: C, 38.10; H, 4.28; N, 22.16%. Anal Found. C, 38.56; H, 4.35; N, 21.82%.

### Synthesis of the coordination compounds

The synthesis of the coordination compounds [Cu(onz)_2_Cl_2_] and [Zn(onz)_2_Cl_2_] was reported previously [16, 17]. However, the reaction conditions were modified to obtain the mononuclear compounds, as discussed below.

#### *[Cu(cenz)*_*2*_*Cl*_*2*_*] (****3****)*

A solution of CuCl_2_**⋅**2H_2_O (45 mg, 0.26 mmol) in acetonitrile (10 mL) was added to a solution of cenz (100 mg, 0.52 mmol) in acetonitrile (5 mL). The reaction mixture was kept under stirring at room temperature for 20 min. After this time a blue precipitate was obtained. The solid product was dissolved in acetonitrile and single crystals were obtained by ether diffusion, which were suitable for X-ray diffraction analysis. Yield: 90%. FT-IR (ATR, cm^−1^): 1560 ν(C=N), 1481 ν_as_(NO_2_), 1425 ν(N–C–N), 1363 ν_s_(NO_2_), 1271 ν(C–N), 1186 ν(N–O). Anal. Calc. for CuCl_4_C_12_H_16_N_6_O_4_: C, 28.05; H, 3.14; N, 16.36%. Anal Found. C, 27.95; H, 3.20; N, 15.86%.

#### *[Cu(cenz)*_*2*_*Br*_*2*_*] (****4)***

A solution of CuBr_2_ (60 mg, 0.26 mmol) in hot ethanol (10 mL) was added to a solution of cenz (100 mg, 0.52 mmol) in hot ethanol (10 mL). The resulting mixture was refluxed for 4 h, after this time the solvent was evaporated under reduced pressure and a brown product was isolated by filtration. Yield: 94%. FT-IR (ATR, cm^−1^): 1548 ν(C=N), 1470 ν_as_(NO_2_), 1435 ν(N–C–N), 1361 ν_s_(NO_2_), 1271 ν(C–N), 1194 ν(N–O). Anal. Calc. for CuBr_2_C_12_H_16_N_6_O_4_Cl_2_: C, 23.92; H, 2.68; N, 13.95%. Anal Found. C, 23.73; H, 2.69; N, 13.68%.

#### *[Zn(cenz)*_*2*_*Cl*_*2*_*] (****5)***

A solution of ZnCl_2_ (36 mg, 0.26 mmol) in hot acetonitrile (10 mL) was added to a solution of cenz 100 mg, 0.52 mmol) in hot acetonitrile (10 mL). The mixture was refluxed for 3 h. The solution was subsequently kept unperturbed for the slow evaporation of the solvent and after two days colorless single crystals, suitable for X-ray diffraction analysis were obtained. Yield: 91%. FT-IR (ATR, cm^−1^): 1554 ν(C=N), 1480 ν_as_(NO_2_), 1420 ν(N–C–N), 1360 ν_s_(NO_2_), 1269 ν(C-N), 1188 ν(N–O). Anal. Calc. for ZnCl_4_C_12_H_16_N_6_O_4_: C, 27.96; H, 3.13; N, 16.30%. Anal Found. C, 27.71; H, 3.47; N, 15.66%.

#### *[Zn(cenz)*_*2*_*Br*_*2*_*] (****6****)*

A solution of ZnBr_2_ (60 mg, 0.26 mmol) in hot acetonitrile (10 mL) was added to a solution of cenz (100 mg, 0.52 mmol) in hot acetonitrile (10 mL). The reaction mixture was refluxed for 3 h, producing a white precipitate that was isolated by filtration. Yield: 88%. FT-IR (ATR, cm^−1^): 1558 ν(C = N), 1478 ν_as_(NO_2_), 1422 ν(N–C–N), 1375 ν_s_(NO_2_), 1269 ν(C–N), 1188 ν(N–O). Anal. Calc. for ZnBr_2_C_12_H_16_N_6_O_4_Cl_2_: C, 23.85; H, 2.67; N, 13.90%. Anal Found. C, 23.49; H, 2.90; N, 13.35%.

#### *[Cu(onz)*_*2*_*Cl*_*2*_*] (****7****)*

A solution of CuCl_2_**·**2H_2_O (43 mg, 0.23 mmol) in hot acetonitrile (10 mL) was added to a solution of onz (100 mg, 0.46 mmol) in hot acetonitrile (10 mL). The resulting mixture was heated stirring for 1 h. Afterwards, the solvent was evaporated, and a viscous product was obtained. A green compound was isolated by precipitation in hexane. Yield: 83%. FT-IR (ATR, cm^−1^): 1556 ν(C=N), 1478 ν_as_(NO_2_), 1425 ν(N–C–N), 1367 ν_s_(NO_2_), 1272 ν(C–N), 1191 ν(N–O). Anal. Calc. for CuCl_4_C_14_H_20_N_6_O_6_: C, 29.31; H, 3.51; N, 14.65%. Anal Found. C, 29.45; H, 3.61; N, 14.86%.

#### *[Cu(onz)*_*2*_*Br*_*2*_*] (****8****)*

A solution of CuBr_2_ (50 mg, 0.23 mmol) in hot acetonitrile (10 mL) was added to a solution of onz (100 mg, 0.46 mmol) in hot acetonitrile (10 mL). The mixture was stirred under heating for 1 h. Afterwards, the solvent was evaporated, and a viscous product was obtained. A brown compound was isolated by precipitation in hexane. Yield: 87%. FT-IR (ATR, cm^−1^): 1556 ν(C=N), 1476 ν_as_(NO_2_), 1425 ν(N–C–N), 1365 ν_s_(NO_2_), 1270 ν(C–N), 1189 ν(N–O). Anal. Calc. for CuBr_2_C_14_H_20_N_6_O_6_Cl_2_: C, 25.38; H, 3.04; N, 12.68%. Anal Found. C, 25.53; H, 3.59; N, 12.40%.

#### *[Zn(onz)*_*2*_*Cl*_*2*_*] (****9****)*

A solution of ZnCl_2_ (31 mg, 0.23 mmol) in hot acetonitrile (10 mL) was added to a solution of onz (100 mg, 0.46 mmol) in hot acetonitrile (10 mL). The mixture was stirred under heating for 1 h. Afterwards, the solvent was evaporated, and a viscous product was obtained. A white compound was isolated by precipitation in hexane. Yield: 82%. FT-IR (ATR, cm^−1^): 1559 ν(C=N), 1480 ν_as_(NO_2_), 1427 ν(N–C–N), 1369 ν_s_(NO_2_), 1272 ν(C–N), 1192 ν(N–O). Anal. Calc. for ZnCl_4_C_14_H_20_N_6_O_6_: C, 29.22; H, 3.50; N, 14.60%. Anal Found. C, 28.99; H, 3.85; N, 14.62%.

#### *[Zn(onz)*_*2*_*Br*_*2*_*] (****10****)*

A solution of ZnCl_2_ (31 mg, 0.23 mmol) in hot acetonitrile (10 mL) was added to a solution of onz (100 mg, 0.46 mmol) in hot acetonitrile (10 mL). The resulting mixture was stirred under heating for 1 h. Afterwards, the solvent was evaporated, and a viscous product was obtained. A white compound was precipitated from hexane. Yield: 81%. FT-IR (ATR, cm^−1^): 1558 ν(C=N), 1480 ν_as_(NO_2_), 1427 ν(N–C–N), 1368 ν_s_(NO_2_), 1270 ν(C–N), 1191 ν(N–O). Anal. Calc. for ZnBr_2_ C_14_H_20_N_6_O_6_Cl_2_: C, 25.31; H, 3.03; N, 12.65%. Anal Found. C, 25.52; H, 3.28; N, 12.80%.

### Physical measurements

Elemental analyses for carbon, hydrogen and nitrogen were carried out with a Perkin Elmer 2400 analyzer. FT IR spectra in the range 4000–400 cm^−1^ and 600–50 cm^−1^ were collected with a Perkin Elmer FTIR/FIR Spectrum 4000 spectrophotometer by attenuated total reflectance (ATR). NMR spectra were obtained at room temperature on a 400 MHz VNMRS Varian with Broad Band Switchable probe of two channel ratio frequency (^1^H/^19^F) (^31^P/^15^N) spectrophotometer of 9.4 T. Electronic spectra were measured over the range 40,000–5000 cm^−1^ by the diffuse reflectance method on a Cary-5000 Varian spectrophotometer at room temperature. Spectra of solution spectra in sodium cacodylate, 3-(*N*-morpholino)-propanesulfonic acid) (MOPS) and phosphate-buffered saline (PBS) were collected in the range of 1600–250 nm. Magnetic susceptibility measurements at room temperature of powdered samples were obtained on a Sherwood Scientific MK1 magnetic susceptibility balance, using the Gouy method.

### X-ray crystallography

X-ray diffraction data for [Cu(cenz)_2_Cl_2_] and [Zn(cenz)_2_Cl_2_] were collected at 130 K using an Oxford Diffraction Gemini “A” diffractometer with a CCD-detector and using graphite monochromated Mo Kα radiation source at 298 K. CrysAlisPro software packages were used for data collection and data integration. Absorption corrections were applied using analytical procedures. The structures were solved by direct methods using the package SHELXS and refined with an anisotropic approach for non-hydrogen atoms using the SHELXL program. All the hydrogen atoms attached to C atoms were positioned geometrically as riding on their parent atoms, with C–H = 0.93–0.99 Å and U_iso_(H) = 1.2 U_eq_(C) for aromatic and methylene groups, and U_iso_(H) = 1.5 U_eq_(C) for methyl groups [34–36]. A summary of the crystallographic data is shown in Table 1. The crystallographic data for the structures have been deposited at the Cambridge Crystallographic Data Centre as supplementary publication CCDC 2293450 and 2272007. Copies of the data can be obtained free of charge via www.ccdc.cam.ac.uk/data_request/cif.

**Table 1:** Crystallographic data of [Cu(cenz)_2_Cl_2_] and [Zn(cenz)_2_Cl_2_]

Compound	[Cu(cenz)_2_Cl_2_]	[Zn(cenz)_2_Cl_2_]
Chemical formula	C_12_H_16_Cl_4_N_6_O_4_Cu	C_12_H_16_Cl_4_N_6_O_4_Zn
FW (g mol^−1^)	513.65	515.48
Crystal system	Orthorhombic	Orthorhombic
Space group	P 2_1_ 2_1_ 2_1_	C m c 2_1_
a (Å)	7.2177(5)	24.725(2) Å
b (Å)	13.5212(8)	7.7268(7) Å
c (Å)	19.9050(11)	9.9383(7) Å
α (°)	90	90
β (°)	90	90
γ (°)	90	90
V (Å^3^)	1942.6(2)	1898.6(3) Å^3^
Z	4	4
D_calc_ (mg cm^−3^)	1.7568	1.803
µ (mm^−1^)	1.707	1.889
F (000)	1036	1040
Temp (K)	130(2)	130(2)
θ range (°)	3.420–29.441	3.440–29.547
Index range	− 8 ≤ h ≤ 9 − 13 ≤ k ≤ 18 − 25 ≤ l ≤ 27	− 31 ≤ h ≤ 34 − 7 ≤ k ≤ 10 − 8 ≤ l ≤ 13
Measured reflection	8007	3038
Parameters	246	128
Final R indices [I > 2sigma(I)]	0.0309	0.0389
wR_2_	0.0579	0.1166
F^2^	1.060	1.067
Δρ_max_ (e Å^−3^)	0.317	1.404
Δρ_min_ (e Å^−3^)	− 0.323	− 1.031

### Biological assays

#### Animals

For the anti-toxoplasma assays three-week-old male CD1 mice (approximate weight 30 g) were obtained from the Faculty of Medicine, UNAM vivarium. The handling of the animals was carried out in accordance with the Mexican Official Norm NOM-062-ZOO-1999 for the production care, and use of laboratory animals in accordance with international guidelines and approved by the Ethical and Research Committee at Faculty of Medicine, UNAM (project 052/2017).

For the acute toxicity assays, young adult male ICR mice with an average weight of 28 g and a variation of no more than ± 3 g were used. The handling of the animals was carried out in accordance with the committee for the care and use of laboratory animals (CICUAL) from the Faculty of Chemistry, UNAM.

#### Tachyzoites of *Toxoplasma gondii*

RH tachyzoites of *Toxoplasma gondii* were extracted in PBS solution at pH 7.4 from intraperitoneal fluid of infected CD1 mice on the third day of infection and centrifuged for ten minutes at 260*g*. Purified parasites were used within 4 h after their isolation.

#### Viability assays in extracellular tachyzoites of *Toxoplasma gondii*

The viability of the parasites was determined from staining with Sytox Green^®^ fluorescent dye exclusion test. Purified tachyzoites (1 × 10^6^) were incubated for one hour at room temperature with PBS solutions of the coordination compounds or the free ligands at concentrations of 40, 20, 10, 5, and 2.5 µM (0.1% DMSO), in a volume final of 1 mL. After the incubation time, the samples were centrifuged for 10 min at 250*g*, the medium was removed and 20 µL of Sytox Green^®^ was added after 15 min of incubation for differentiate dead parasites in a Neubauer chamber. The assays were performed in triplicate. The half maximal inhibitory concentration (IC_50_) was calculated by Boltzmann regression analysis and expressed as mean ± standard error of three replicates. Based on the results the tachyzoites were incubated under the same conditions with the coordination compounds [Cu(onz)_2_Cl_2_] and [Cu(onz)_2_Br_2_] at their respective IC_50_ values, Sytox Green^®^ was then added, and they were observed by fluorescence microscopy. Sulfadiazine was used as positive control.

#### Pharmacological profile and acute toxicity

A stock solution with a concentration of 25 mg mL^−1^ of [Cu(onz)_2_Cl_2_] and [Cu(onz)_2_Br_2_] was prepared with water for injection. In the case of [Cu(onz)_2_Br_2_], 0.1% of DMSO was added. For the pharmacological profile three mice were used for each dose of 1, 10 and 100 mg kg^−1^ per compound. For the acute toxicity assays five mice were treated for each dose (10, 50, 75 and 100 mg kg^−1^) per compound. All study animals were observed at least twice daily to determine pain/mortality. A detailed clinical observation was made 24 h after administration. The doses were administered intraperitoneally. The data obtained were used to calculate the mean lethal dose using the software Prism 13.0 and SPSS 29.0 [37, 38].

### *Distribution coefficient (log D*_*7.4*_*)*

Solutions between 100 and 10 µM of the free ligands (cenz and onz) and the coordination compounds 3–10 were prepared in a MOPS buffer pH 7.4 as the aqueous phase and in 1-octanol as the organic phase. The absorption at 320 nm and 310 nm was determined for the aqueous and organic phase respectively by electronic spectroscopy (UV–Vis). Through a linear regression (concentration vs. absorption) the molar extinction coefficient of each compound in both phases was determined (Table 3). The lipophilicity was calculated in the 1-octanol/water system from the shake flask method using Eq. 1 [39]:

1 $$Log{D}_{7.4}=\frac{{C}_{oc}}{{C}_{w}}$$

where, C_o_ is the final concentration in the organic phase and C_w_ is the final concentration in the aqueous phase (MOPS buffer at 10^–2^ M pH 7.4). Both phases were presaturated with 50 µM solutions of the free ligands or 500 µM of each coordination compound and their UV spectrum was recorded. Both phases were combined and kept stirring for 3 h at 25 °C and in the absence of light. Subsequently, the mixture was centrifuged at 400*g* and the concentration in both phases was determined by UV–Vis spectroscopy using the Beer-Lambert law. The assays were performed in triplicate.

### Studies on DNA interaction and damage

For the UV–Vis, fluorescence and circular dichroism assays the concentration of ct-DNA was determined on a Varian Cary 100 Scan spectrophotometer from the absorption at 260 nm and the corresponding molar absorptivity of 6600 M^−1^ cm^−1^ (nucleobase concentration). The stock solutions of the coordination compounds and the free ligands were prepared in cacodylate/NaCl buffer (1 mM cacodylic acid and 20 mM sodium chloride) at pH 7.25. The final samples contained a DMSO concentration of 0.2%.

#### UV–Vis measurements

Absorption titration experiments were carried out by adding increasing concentrations of ct-DNA (0–25 µM) to 10 µM of the compounds in a final volume of 2 mL. The samples were incubated for 1 h at 37 °C, and spectra were collected on a Varian Cary 100 Scan spectrophotometer. For these assays the charge-transfer band of the compounds was followed at 320 nm. The intrinsic binding constant (K_b_) was determined from the titration data using Eq. 2:

2 $$\frac{\left[DNA\right]}{{\varepsilon }_{\alpha }-{\varepsilon }_{f}}=\frac{\left[DNA\right]}{{\varepsilon }_{0}-{\varepsilon }_{f}}+\frac{1}{{K}_{b}\left({\varepsilon }_{0}-{\varepsilon }_{f}\right)}$$

where ε_a_, ε_0_ and ε_f_ are respectively, the molar extinction coefficients of the free compounds in solution, fully bound compound with DNA and compound bound to DNA at a definite concentration.

#### Fluorescence spectroscopy

Emission intensity of the dyes DNA-intercalator ethidium bromide, EB (λ_exc_ = 514 nm) and the minor-groove binder Hoechst 33,258 (λ_exc_ = 350 nm) was measured on iHR320 HORIBA JOBIN YVON spectrofluorometer. The samples contained 15 µM (in base pairs) of ct-DNA and 75 µM of EB or Hoechst 33,258 in cacodylate/NaCl buffer. The respective dye was incubated for 30 min with ct-DNA to allow its binding to the biomolecule. Subsequently, increasing amounts of coordination compounds (0–50 µM) were added up to a final volume of 2 mL, and the samples were incubated for 1 h at 37 °C. The fluorescence spectra of all samples were recorded at room temperature. The affinity of the compounds for ct-DNA compared to EB was evaluated through the Stern–Volmer quenching constant K_sv_, from Eq. 3:

3 $$\frac{{I}_{0}}{I}=1+{K}_{sv}\left[compound\right]$$

where I_0_ and I are the emission intensities in absence and the presence of the compound, respectively.

#### Circular dichroism (CD)

Circular dichroism (CD) spectra were recorded using a JASCO-815 spectropolarimeter, equipped with xenon-arc lamp of 450 W. The sample compartment was air-purged with N_2_ before use. For the measurements, a quartz cuvette with an optical path of 5 mm was used. A solution of 25 µM (in base pairs) of ct-DNA in cacodylate/NaCl buffer was incubated for 1 h at 37 °C with increasing amounts of coordination compounds (0–50 µM). The CD spectra were recorded at room temperature.

#### Agarose gel electrophoresis

Electrophoresis assays were performed in 1.5% agarose gel and 1× TBE buffer using the DNA plasmid pBR322. The plasmid (15 µM base pairs) was incubated with 10 and 50 μM concentrations of the coordination compounds for 1 h at 37 °C in presence and absence of ascorbic acid (50 μM) as reducing agent and hydrogen peroxide (500 µM) as ROS generator in a final volume of 20 μL. The DNA-cleaving compound [Cu(phen)_2_(H_2_O)](NO_3_)_2_ was used as reference. The samples were loaded into the gel with 4 μL of a loading buffer (30% glycerol, 5 mM xylene cyanol), and electrophoresis was performed in 1× TBE at 100 V for 1 h in a Bio-Rad horizontal tank. Subsequently, the gel was stained with SYBR™ Safe overnight and images were acquired with a Gel Doc EZ Imager instrument (Bio-Rad).

## Results and discussion

### Spectroscopic characterization

The chemical structures of the coordination compounds were proposed based on spectroscopic data as well as elemental analyses and effective magnetic moment.

#### IR spectra

The vibration band ν(C=N) presents a significant shift, which is indicative of the ligand coordination to the metal center through the aromatic nitrogen. This vibration is observed at 1520 cm^−1^ for free cenz and at 1548–1560 cm^−1^ for its compounds. For the onz ligand, this vibration at 1536 cm^−1^ is shifted in the coordination compounds at 1552–1559 cm^−1^. The shifting of the ν(NCN) and ν(C–N) vibration bands corroborate the coordination of the metal ion in the range of 1425–1476 cm^−1^ and 1270–1274 cm^−1^, respectively. The asymmetric vibration band of the nitro group ν(NO_2_)_as_ is observed at 1458 and 1466 cm^−1^ for cenz and onz respectively, which is shifted for more than 12 cm^−1^ in their coordination compounds due to non-covalent interactions, as observed from the X-ray structures of [Zn(cenz)_2_Cl_2_] and [Cu(cenz)_2_Cl_2_].

Additionally, in the far infrared region of spectra, metal-halogen vibration ν(M-X) bands were observed. These coordination compounds showed the two normal modes of vibration (a_1_ + b_1_) allowed for a tetrahedral geometry [40]. The assignments for each compound are shown in Table 2.

**Table 2:** Far infrared bands (cm^−1^) and effective magnetic moments (BM) for the coordination compounds

Compound	ν(M-Cl)	µ_eff_	Compound	ν(M-Br)	µ_eff_
[Cu(cenz)_2_Cl_2_]	326, 276	2.07	[Cu(cenz)_2_Br_2_]	199	1.86
[Zn(cenz)_2_Cl_2_]	332, 296	–-	[Zn(cenz)_2_Br_2_]	270, 214	–-
[Cu(onz)_2_Cl_2_]	317, 273	1.78	[Cu(onz)_2_Br_2_]	224, 185	1.99
[Zn(onz)_2_Cl_2_]	331, 273	–-	[Zn(onz)_2_Br_2_]	275, 249	–-

#### Electronic spectroscopy and magnetic moments

The diffuse reflectance spectra for the copper(II) complexes [Cu(onz)_2_Cl_2_] and [Cu(onz)_2_Br_2_] showed a multicomponent electronic transition band, centered at 12,560 and 12,450 cm^−1^, respectively, characteristic of a pseudo-tetrahedral geometry [41]. Similar bands were observed for [Cu(cenz)_2_Cl_2_] and [Cu(cenz)_2_Br_2_] compounds at 12,580 and 12,140 cm^−1^, respectively. The charge transfer bands were centered at 27,530 and 24,750 cm^−1^ for [Cu(onz)_2_Cl_2_] and [Cu(onz)_2_Br_2_], while for [Cu(cenz)_2_Cl_2_] and [Cu(cenz)_2_Br_2_], they were observed at 23,230 and 22,800 cm^−1^, respectively (Fig. S2).

The effective magnetic moments for [Cu(cenz)_2_Cl_2_], [Cu(cenz)_2_Br_2_], [Cu(onz)_2_Cl_2_] and [Cu(onz)_2_Br_2_] are in the range expected for such copper(II) coordination compounds, Table 2 [42].

#### Solution stability assays

The solution spectra for the copper(II) coordination compounds were obtained in the buffer used for the distribution coefficient studies (namely, MOPS buffer at 10^–2^ M pH 7.4), the determination of the antiparasitic activities (PBS) and the DNA-damage studies (cacodylate 1 mM / NaCl 20 mM pH 7.25). The spectra were obtained from complex solution of 5 mM, in a time interval between 0 and 24 h. During this period, a band centered between 800 nm (12,500 cm^−1^) and 810 nm (12,345 cm^−1^) was observed (Fig. S3), indicative that the pseudo-tetrahedral geometry is preserved in solution under physiological conditions.

The charge-transfer bands of the studied compounds were used for the distribution coefficient assays and the DNA-damage studies, which were carried out with UV–Vis spectroscopy, at 320 nm in buffer solutions and at 310 nm in octanol. These bands were also used for the partition assays with a complex concentration of 100 µM, in MOPS buffer (10^–2^ M pH 7.4) and in octanol.

#### NMR

For the zinc(II) compounds, [Zn(cenz)_2_Cl_2_], [Zn(cenz)_2_Br_2_], [Zn(onz)_2_Cl_2_] and [Zn(onz)_2_Br_2_], the ^1^H NMR spectra were recorded in DMSO-d_6_.

The coordination of the 5-nitroimidazole imidazole nitrogen atom to the metal center was corroborated by the shifting of the H4 aromatic proton signal. For the free cenz and onz ligands, the H4 signal is at 8.02 and 7.03 ppm, respectively, and shifts to higher ppm values upon coordination to zinc(II) (Fig. S4, S5).

### X-ray structures

**Fig. 2:**
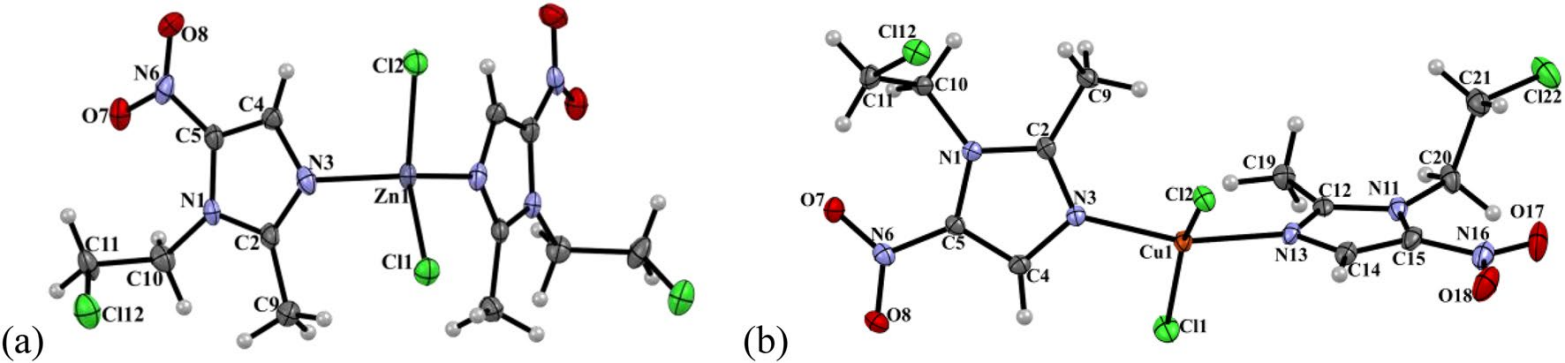
ORTEP representation of **a** [Zn(cenz)_2_Cl_2_] and **b** [Cu(cenz)_2_Cl_2_] with ellipsoids at 50% probability

[Zn(cenz)_2_Cl_2_] crystallized in the orthorhombic crystal system, Cmc2_1_ space group. In this compound two 5-nitroimidazole ligands are coordinated to the metal center through the imidazolic N3 atom, which are reflected through a plane of symmetry, and two chloride ligands complete the coordination sphere (Fig. 2). The angles around the metal center have values between 103.45 (15)° and 122.45 (10)°. The calculated parameter τ_4_ = 0.9 is indicative of a tetrahedral geometry [43]. Based on the spectroscopic data, a similar tetrahedral geometry for the zinc(II) compounds [Zn(cenz)_2_Br_2_], [Zn(onz)_2_Cl_2_] and [Zn(onz)_2_Br_2_] is expected.

The compound [Cu(cenz)_2_Cl_2_] crystallizes in an orthorhombic crystal system with P2_1_2_1_2_1_ space group. The coordination sphere is similar to that of the zinc(II) compound describe above, but in this case the parameter τ_4_ = 0.32, which is indicative of a distorted tetrahedral geometry, as reflected by the angles of ca. 91.64(8)° and 158.8(1)°. For [Cu(cenz)_2_Br_2_], [Cu(onz)_2_Cl_2_] and [Cu(onz)_2_Br_2_], a similar coordination geometry is proposed, based on their comparable spectroscopic data.

**Fig. 3:**
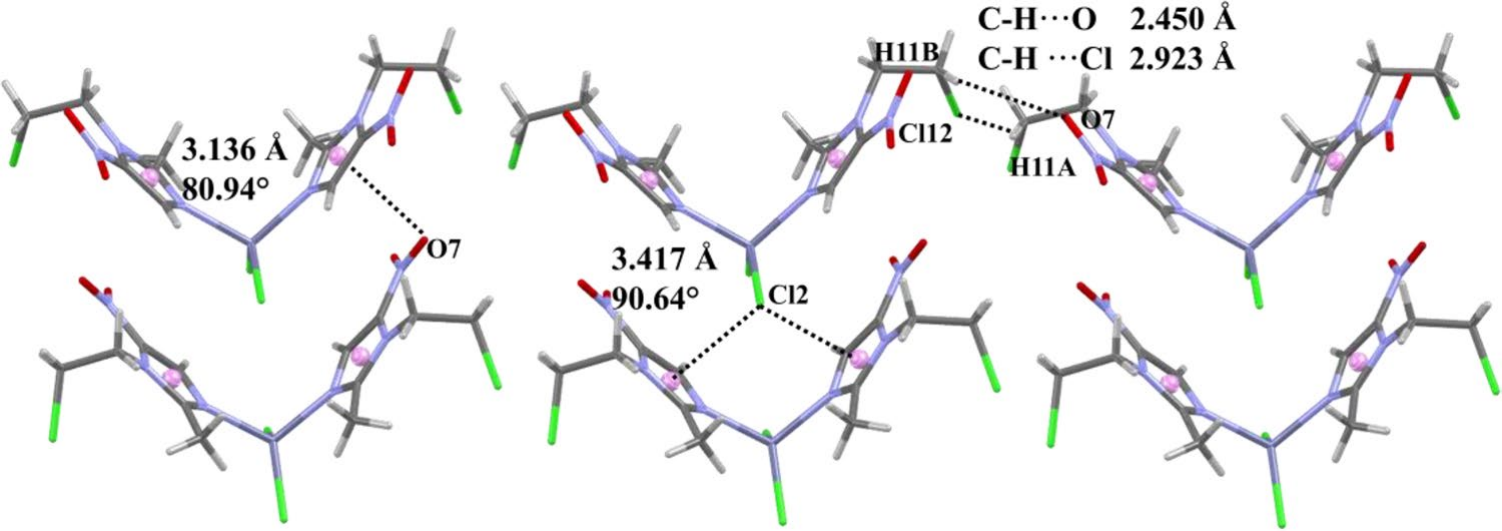
Non-covalent interactions observed for [Zn(cenz)_2_Cl_2_] in its solid-state structure

The crystal packing of [Zn(cenz)_2_Cl_2_] is stabilized by lone pair∙∙∙π interactions (Fig. 3), between one of the coordinated halogen atoms and the π system of the imidazole ring, with Zn-Cl2∙∙∙π_iz_ (Cl∙∙∙centroid) = 3.417 Å and an angle of 90.64°. Furthermore, the nitro group is also involved in lone pair∙∙∙π interactions, with N6-O7∙∙∙π_iz_ = 3.136 Å and angle of 80.94°. Finally, hydrogen bonds are observed between the nitro group and the ligand alkyl chains, viz*.* ONO∙∙∙H-C11 = 2,45 Å, and Cl12∙∙∙H-C11A = 2.923 Å.

**Fig. 4:**
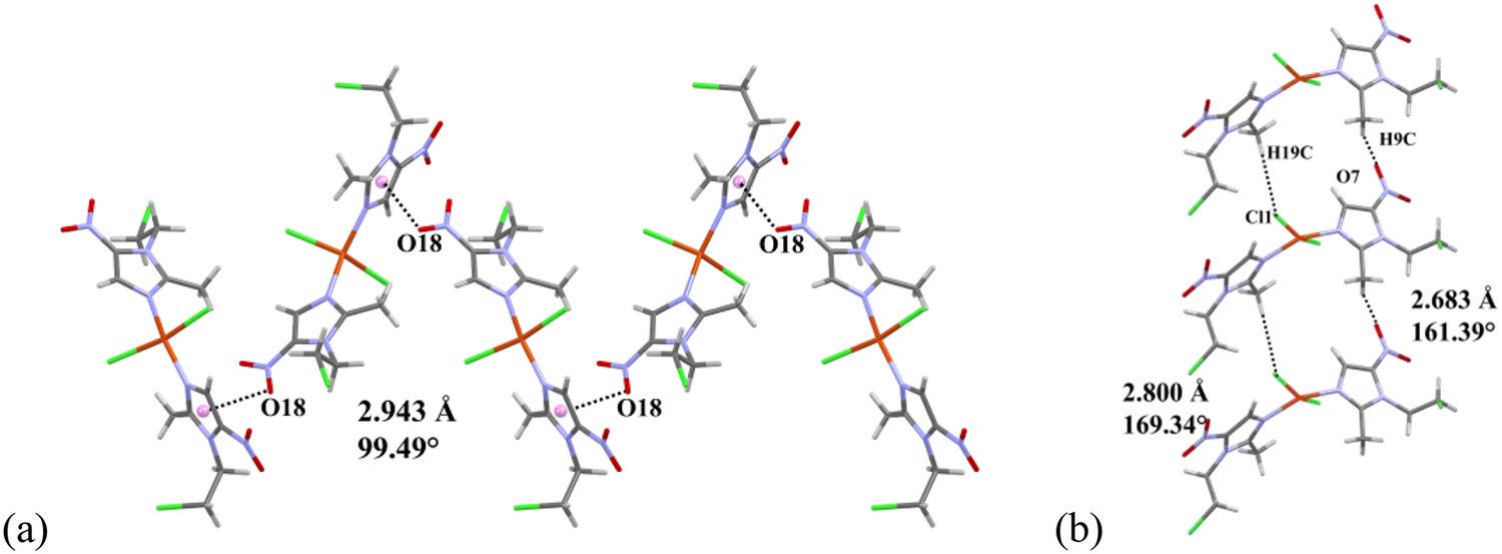
**a** Lone pair∙∙∙π interactions and **b** hydrogen bonds observed in the solid-state structure of [Cu(cenz)_2_Cl_2_]

For [Cu(cenz)_2_Cl_2_], lone pair∙∙∙π interactions take place between the nitro group and a neighboring imidazolic ring (ON-O18∙∙∙π_iz_ = 2.943 Å), which stabilize the crystal packing of the molecule. Hydrogen bonds are also observed between the methyl group and one of the coordinated chlorido ligand (C19-H19C···Cl1 = 2.800 Å), as well as with the nitro group (C9-H9C···O7 = 2.683 Å) (Fig. 4).

### Anti-toxoplasma activity

Viability assays were performed against tachyzoites of *Toxoplasma gondii*. The RH strain was used to evaluate the effect of different concentrations (namely, 2.5, 5, 10, 20 and 40 µM) of the coordination compounds and the free 5-nitroimidazole against the tachyzoites. The evaluation were performed in PBS buffer (pH 7.4) with Sytox Green^®^ exclusion test, which allows differentiating dead from live tachyzoites, since the intact cell membrane of alive ones is not stained [44].

**Fig. 5:**
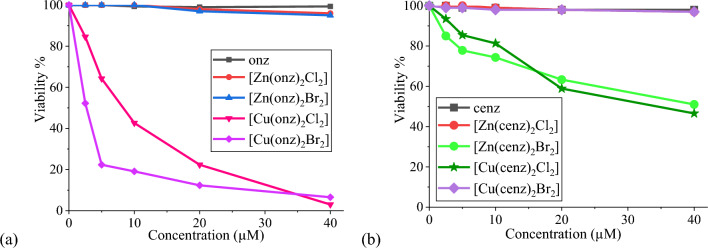
Viability (%) of *Toxoplasma gondii* (RH tachyzoites) exposed to **a** onz and **b** cenz and their coordination compounds (0–40 µM) for 1 h in PBS buffer

The free cenz and onz ligands did not show activity against *T. gondii*. The copper(II) compounds [Cu(onz)_2_Cl_2_] and [Cu(onz)_2_Br_2_] showed IC_50_ values of 8.49 ± 0.64 and 2.73 ± 0.34 µM, respectively, thus these two complexes are more active than sulfadiazine (IC_50_ > 1600 µM), one of the drugs of choice against toxoplasmosis. [Zn(cenz)_2_Br_2_] was the only zinc(II) compound that presented activity, with a IC_50_ value of 40.0 ± 0.96 µM. Finally [Cu(cenz)_2_Cl_2_] was also active (IC_50_ = 30.55 ± 0.25 µM). The plots of viability percent vs. the concentration of the tested compounds are presented in Fig. 5. The viability plots obtained by Boltzmann regression analysis for the active compounds and the sulfadiazine are shown in the supplementary material (Fig. S6).

**Fig. 6:**
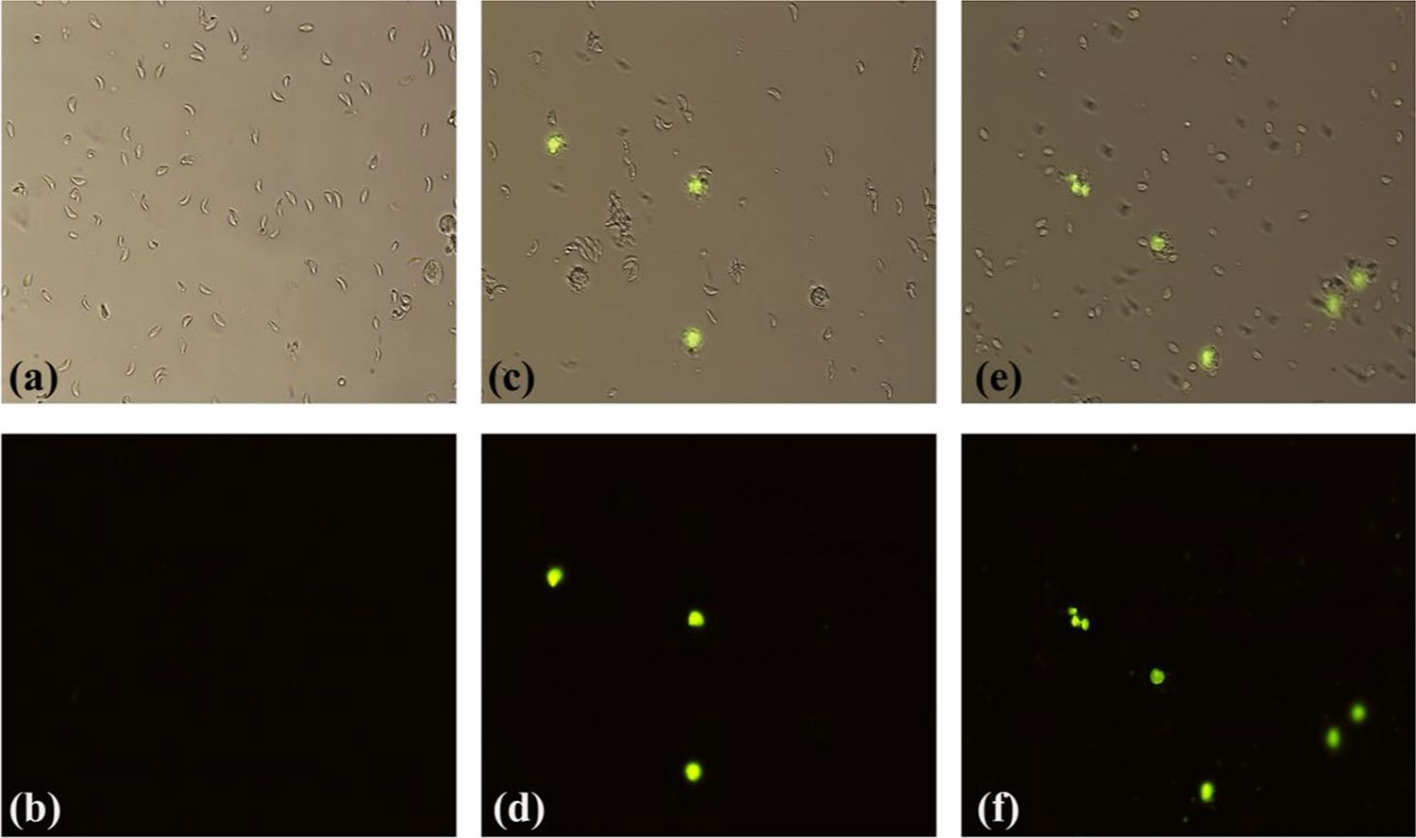
Images of *T. gondii* RH strain tachyzoites: **a** control without treatment in bright field and **b** in fluorescence microscopy; **c** and **d** exposed to [Cu(onz)_2_Cl_2_] (8 µM); **e** and **f** exposed to [Cu(onz)_2_Br_2_] (3 µM)

To corroborate the toxic effect of [Cu(onz)_2_Cl_2_] and [Cu(onz)_2_Br_2_], assays were carried out by fluorescence microscopy, using Sytox^®^ Green, a nucleic acid stain that allows distinguishing dead cells, by becoming fluorescent when entering the damaged membrane and binding to the DNA [26]. Fluorescent images for [Cu(onz)_2_Cl_2_] and [Cu(onz)_2_Br_2_] are shown in Fig. 6.

In order to investigate the effect on host cells of the most active coordination compounds against *T. gondii*, the cytotoxic activity in healthy mouse fibroblast L929 cells was determined. The IC_50_ values obtained for [Cu(onz)_2_Cl_2_] (210.2 μM) and [Cu(onz)_2_Br_2_] (180.2 μM) showed low toxicity in healthy cells compared to the great anti-toxoplasma activity they presented.

### *Acute toxicity (LD*_*50*_*)*

Based on the anti-toxoplasma results the medial lethal dose (*LD*_*50*_) for [Cu(onz)_2_Cl_2_] and [Cu(onz)_2_Br_2_] were determined intraperitoneally using doses of 10, 50, 75 and 100 mg kg^−1^ per compound in young adult male ICR mice.

The LD_50_ values were 49.35 and 83.24 mg kg^−1^ for [Cu(onz)_2_Cl_2_] and [Cu(onz)_2_Br_2_], respectively. The value reported for sulfadiazine (through intraperitoneal injection) is 180 mg kg^−1^ [45]. The coordination compounds appear to be more active than the standard drug. Considering these highly promising preliminary LD_50_ results (toxicity at a single dose) additional studies with these coordination compounds may be envisaged.

### Distribution coefficient

The *T gondii* parasite is an apicomplex organism; it requires a host cell to live, so it is important that the drugs used against the parasite have easy access to the cell. The lipophilicity of a compound is an important physicochemical property to determine its ability to permeate the biological membrane through passive diffusion; which usually is measured from the distribution coefficient logD_7.4_ = log(C_o_/C_w_), in n-octanol/water [39]. The shake-flask method was used to determine the LogD_7.4_ values from the molar extinction coefficients of the coordination compounds and the ligands in the two solvents (Table 3). For all compounds, positive LogD_7.4_ values were obtained, indicating that they are capable of crossing the cell membrane via passive diffusion, due to a good balance between permeability and solubility [39].

**Table 3:** Molar extinction coefficients in octanol and water (MOPS buffer 10^–2^ M pH 7.2) for the ligands and the coordination compounds and corresponding LogD_7.4_ (logarithm of distribution ratio octanol/water) values

Compound	ε_oc_ (M^−1^ cm^−1^) λ_max_ = 320 nm	ε_buffer_ (M^−1^ cm^−1^) λ_max_ = 310 nm	LogD_7.4_
onz	7945.45 ± 25.43	9702.98 ± 67.69	0.64
[Cu(onz)_2_Cl_2_]	1296.36 ± 20.70	1366.66 ± 30.00	0.61
[Cu(onz)_2_Br_2_]	1359.36 ± 45.80	1489.64 ± 15.80	0.56
[Zn(onz)_2_Cl_2_]	1274.74 ± 35.40	1036.86 ± 16.50	0.90
[Zn(onz)_2_Br_2_]	1289.53 ± 41.79	1044.84 ± 14.25	0.95
cenz	8236.23 ± 46.50	9846.14 ± 16.50	0.72
[Cu(cenz)_2_Cl_2_]	1246.51 ± 15.58	1346.55 ± 45.35	0.69
[Cu(cenz)_2_Br_2_]	1365.65 ± 13.26	1465.15 ± 46.56	0.62
[Zn(cenz)_2_Cl_2_]	1249.85 ± 32.89	1194.46 ± 16.50	0.86
[Zn(cenz)_2_Br_2_]	1246.56 ± 34.50	1148.69 ± 34.55	0.82

### DNA-damage

The interaction of small molecules with DNA has been studied extensively through different spectroscopic techniques with the aim of understanding the possible mechanisms of action of potential drugs. The interaction with DNA may be of electrostatic nature, for instance between the negatively charged phosphate backbone of DNA and the positively charged ends of small molecules. Groove binding involves hydrogen or van der Waals interactions between small molecules and nucleic bases within the major or minor groove. The DNA-interaction may also occur through intercalation of the molecules between nucleic base pairs [46, 47].

#### UV–Vis spectroscopy

UV–Vis spectroscopy is one of the most effective methods to study the interaction of small molecules with DNA. In the present case the absorption maxima of the coordination compounds around 320 nm, exhibit changes upon the addition of increasing amounts of ct-DNA (1 h of incubation).

**Fig. 7:**
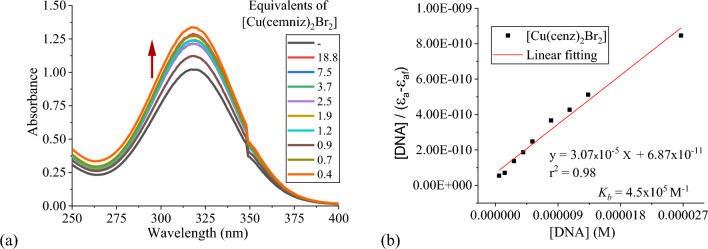
**a** Absorption spectra of 10 mM solution of [Cu(cenz)_2_Cl_2_] upon increasing [ct-DNA] (0–27 mM), in cacodylate buffer (pH 7.25) and applying an incubation time of 1 h, **b** Linear fitting of plots [DNA]/(ε_a_ − ε_f_) vs [DNA] for the titration of ct-DNA

In general, the copper(II) coordination compounds present a hyperchromic effect when [ct-DNA] increases, while for the zinc(II) compounds, a hypochromic effect, is observed. For all the complexes investigated no wavelength shift was observed. This behavior is characteristic of groove binding or electrostatic interactions with DNA. From Eq. 2 the binding constant (*K*_*b*_) was determined (Table 4). Higher values are obtained for the copper(II) compounds, indicating a stronger DNA binding than the zinc(II) compounds (Fig. 7).

**Table 4:** *K*_*b*_ binding constant (UV–Vis) and Stern–Volmer quenching constant *K*_*sv*_ for the copper(II) and zinc(II) coordination compounds using EB (75 µM) and Hoechst 33258 (1.9 µM); [DNA]_b_ = 30 µM

Coordination compound	*K*_*b*_ (M^−1^)	Ethidium bromide *K*_*sv*_ (M^−1^)	Hoechst 33258 *K*_*sv*_ (M^−1^)	Coordination compound	*K*_*b*_ (M^−1^)	Ethidium bromide *K*_*sv*_ (M^−1^)	Hoechst 33258 *K*_*sv*_ (M^−1^)
[Cu(onz)_2_Cl_2_]	1.2 × 10^5^	2.6 × 10^4^	1.2 × 10^6^	[Cu(cenz)_2_Cl_2_]	7.3 × 10^4^	–	1.6 × 10^6^
[Cu(onz)_2_Br_2_]	4.2 × 10^5^	4.3 × 10^4^	2.5 × 10^6^	[Cu(cenz)_2_Br_2_]	4.5 × 10^5^	3.5 × 10^4^	2.1 × 10^6^
[Zn(onz)_2_Cl_2_]	3.5 × 10^4^	4.5 × 10^3^	3.8 × 10^4^	[Zn(cenz)_2_Cl_2_]	2.3 × 10^4^	–	6.1 × 10^4^
[Zn(onz)_2_Br_2_]	4.2 × 10^4^	2.6 × 10^3^	4.8 × 10^4^	[Zn(cenz)_2_Br_2_]	4.7 × 10^4^	1.1 × 10^3^	1.2 × 10^5^

#### Competitive displacement assays using fluorescent DNA-binders

With the aim to corroborate the binding mode of the coordination compounds with DNA competitive displacement assays were carried out by florescence spectroscopy. The fluorescence of ethidium bromide (EB) increases when it intercalates between DNA base pairs, forming an EB-DNA adduct. Competitive binding assays will lead to a decrease of the fluorescence intensity (λ_exc_ = 514 nm; λ_em_ = 610 nm) if the interacting molecule is capable of displacing EB from the DNA helix [48]. Solutions containing constant concentrations of DNA (30 µM) and EB (75 µM) were incubated for 30 min. Then the coordination compounds to be tested were added in a concentration in the range of 0–50 µM (corresponding to [complex]/[DNA] ratios between = 0 and 3.3), and fluorescence spectra were recorded after 1 h of incubation.

**Fig. 8:**
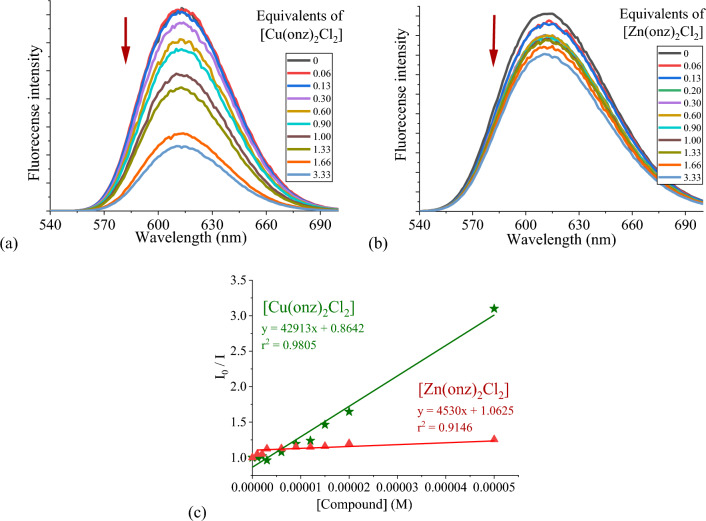
Competitive displacement assays for the DNA-EB adducts, upon addition of increasing amounts of **a** [Cu(onz)_2_Cl_2_] and **b** [Zn(onz)_2_Cl_2_]; **c** Stern–Volmer plots of I_0_/I vs. [compound] for the titration of DNA-EB. Experimental data points and linear fitting after 1 h of incubation. [EB] = 75 µM, [DNA]_b_ = 30 µM, [compound] = 0–50 µM, λ_exc_ = 514 nm and λ_em_ = 610 nm

In all cases fluorescence quenching was observed; the spectra obtained for [Cu(onz)_2_Cl_2_] and [Zn(onz)_2_Cl_2_] are shown in Fig. 8. This behavior is not necessarily due to DNA intercalation. Electrostatic interactions or groove binding may be strong enough to modify the DNA double helix and displace EB. From the slope of the plot of I_0_/I values vs concentration (Fig. 8c), the Stern–Volmer *K*_*sv*_ values for each compound were obtained (Table 4). These values characterize a moderate EB-displacement, the copper(II) compounds being more effective that the zinc(II) ones. For [Cu(cenz)_2_Cl_2_] and [Zn(cenz)_2_Cl_2_], it was not possible to determine *K*_*sv*_ because the plot was non-linear.

**Fig. 9:**
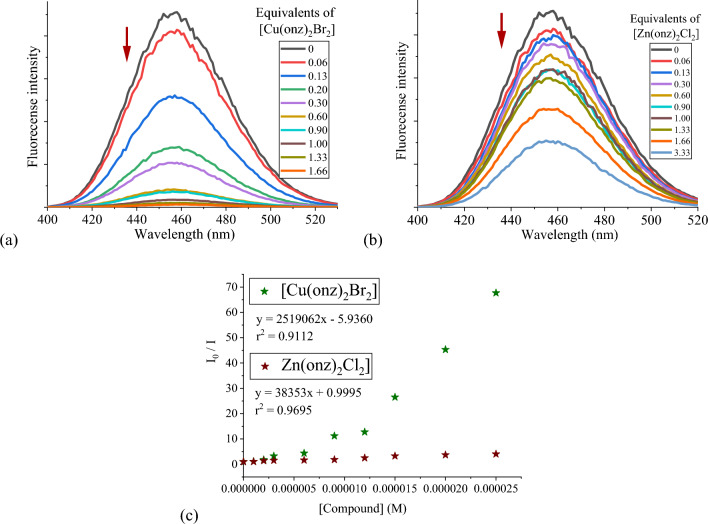
Competitive displacement assays for the DNA-Hoechst 33258 adduct, upon addition of increasing amounts of **a** [Cu(onz)_2_Br_2_] and **b** [Zn(onz)_2_Cl_2_]; **c** Stern–Volmer plots of I_0_/I vs. [compound] for the titration of DNA-Hoechst 33258. Experimental data points and linear fitting after 1 h of incubation. [Hoechst 33258] = 1.9 µM, [DNA]_b_ = 30 µM, [compound] = 0–50 µM, λ_exc_ = 350 nm and λ_em_ = 458 nm

Competitive binding assays were carried out with Hoechst 33,258. This dye binds to B-DNA, especially those with an AT-rich sequence giving a fluorescent signal at 458 nm when excited at 350 nm [49]. As for to the EB assays, DNA (30 µM) was incubated for 30 min with Hoechst 33,258 (1.9 µM) and increasing amounts of the coordination compounds were subsequently added. In all cases, fluorescence quenching was observed (Fig. 9). As previously, the *K*_*sv*_ constants were determined (Fig. 9c and Table 4). The copper(II) compounds show *K*_*sv*_ values in the 1.2–2.5 × 10^–6^ M^−1^ range (Table 4), indicating a groove binding behavior. For the zinc(II) compounds, the *K*_*sv*_ values are two orders of magnitude lower indicating weaker interactions, must likely of electrostatic nature.

#### Circular dichroism (CD)

Circular dichroism (CD) spectroscopy was used to analyze the conformational changes of ct-DNA, its spectrum exhibited a characteristic positive band at 275 nm due to base stacking interactions, and a negative band at 245 nm originating from the right-handed helicity of DNA B-form [50, 51]. The interaction of small molecules with DNA can modify these CD signals depending on the type of binding. Intercalative interactions increase the intensity of the signals, as the result of an increase of the stability of the B conformation, whereas groove binding or covalent interactions tend to decrease the intensity of these signals, which are moreover displaced [51, 52]. The CD spectra for both ligands and their copper(II) and zinc(II) complexes were obtained at constant concentration of ct-DNA, increasing the complex concentration up to two equivalents ([complex]/[DNA] ratios of 0, 0.2, 0.4, 1, 2) with respect to the nucleobases concentration ([DNA]_b_ = 50 µM).

**Fig. 10:**
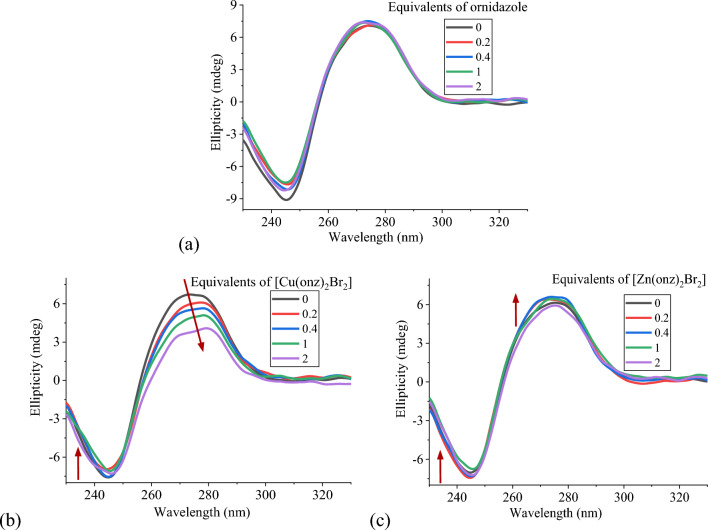
CD spectra of ct-DNA (50 µM_base_) in the presence of increasing amounts of **a** onz, **b** [Cu(onz)_2_Br_2_] and **c** [Zn(onz)_2_Br_2_], in 1 mM cacodylate/20 mM NaCl buffer (pH 7.25). The spectra were recorded after 1 h incubation at 37 °C

The free ligands cenz and onz did not affect the DNA structure, while the increase of the concentration of [Cu(cenz)_2_Cl_2_], [Cu(cenz)_2_Br_2_], [Cu(onz)_2_Cl_2_] or [Cu(onz)_2_Br_2_], give rise to a decrease of the intensity of the positive and negative bands at 275 nm and 245 nm, accompanied by a bathochromic shift. This phenomenon is indicative of alteration of the base stacking interactions and the helicity, which may be explained by the groove binding of complexes [53]. For [Zn(cenz)_2_Cl_2_], [Zn(cenz)_2_Br_2_], [Zn(onz)_2_Cl_2_] and [Zn(onz)_2_Br_2_], the spectra changes are minor, which can be expected for compounds undergoing electrostatic interactions with the biomolecule (see aboce) which do not significantly modify its structure. CD spectra for onz coordination compounds and its copper(II) and zinc(II) complexes are shown at in Fig. 10.

### Agarose gel electrophoresis

Distinct forms of pBR322 plasmid DNA are commonly observed by gel electrophoresis due to the conformational changes that present distinct electrophoretic mobility: (1) supercoiled DNA (Form I) that migrates faster on the gel, (2) circular nicked (Form II), obtained from a one-strand scission, has a slower migration through the gel and (3) linear form (Form III) is generated when both strands are cleaved and migrates between forms I and II [54]. The interaction of the free ligands and their copper(II) and zinc(II) coordination compounds with pBR322 was investigated in presence of H_2_Asc (50 μM) as reducing agent and H_2_O_2_ (500 μM) as ROS generator. Solutions of 10 and 50 µM of the coordination compounds were incubated with the DNA at 37 °C for 1 h.

Solely, the data achieved with cenz as ligand are described below, the results obtained with onz being comparable (see Tables S1-S4).

**Fig. 11:**
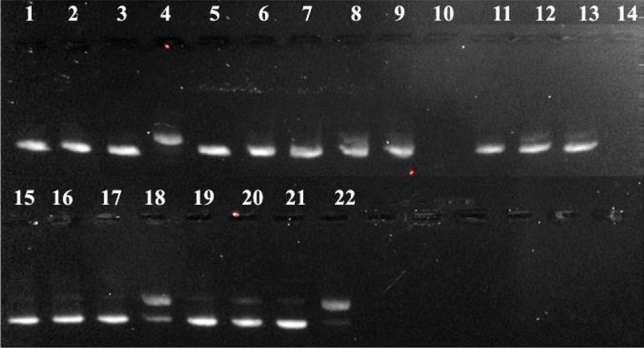
Agarose gel electrophoresis image of 15 µM_pb_ pBR322 plasmid DNA incubated for 1 h at 37 °C with 10 and 50 µM concentrations of cenz coordination compounds in cacodylate-NaCl buffer pH 7.2. Lane 1-plasmid DNA (p); lane 2-p + H_2_Asc; lane 3-p + [Cu(phen)(H_2_O)]^+^; lane 4-p + [Cu(phen)(H_2_O)]^+^  + H_2_Asc; lane 5-p + cenz; lane 6-p + cenz + H_2_Asc; lane 7-p + [Cu(cenz)_2_Cl_2_] (10 µM); lane 8-p + [Cu(cenz)_2_Cl_2_] (10 µM) + H_2_Asc; lane 9-p + [Cu(cenz)_2_Cl_2_] (50 µM); lane 10-p + [Cu(cenz)_2_Cl_2_] (50 µM) + H_2_Asc; lane 11-p + [Cu(cenz)_2_Br_2_] (10 µM); lane 12-p + [Cu(cenz)_2_Br_2_] (10 µM) + H_2_Asc; lane 13-p + [Cu(cenz)_2_Br_2_] (50 µM); lane 14-p + [Cu(cenz)_2_Br_2_] (50 µM) + H_2_Asc; lane 15-p + [Zn(cenz)_2_Cl_2_] (10 µM); lane 16-p + [Zn(cenz)_2_Cl_2_] (10 µM) + H_2_Asc; lane 17-p + [Zn(cenz)_2_Cl_2_] (50 µM); lane 18-p + [Zn(cenz)_2_Cl_2_] (50 µM) + H_2_Asc; lane 19-p + [Zn(cenz)_2_Br_2_] (10 µM); lane 20-p + [Zn(cenz)_2_Br_2_] (10 µM) + H_2_Asc; lane 21-p + [Zn(cenz)_2_Br_2_] (50 µM); lane 22-p + [Zn(cenz)_2_Br_2_] (50 µM) + H_2_Asc

**Fig. 12:**
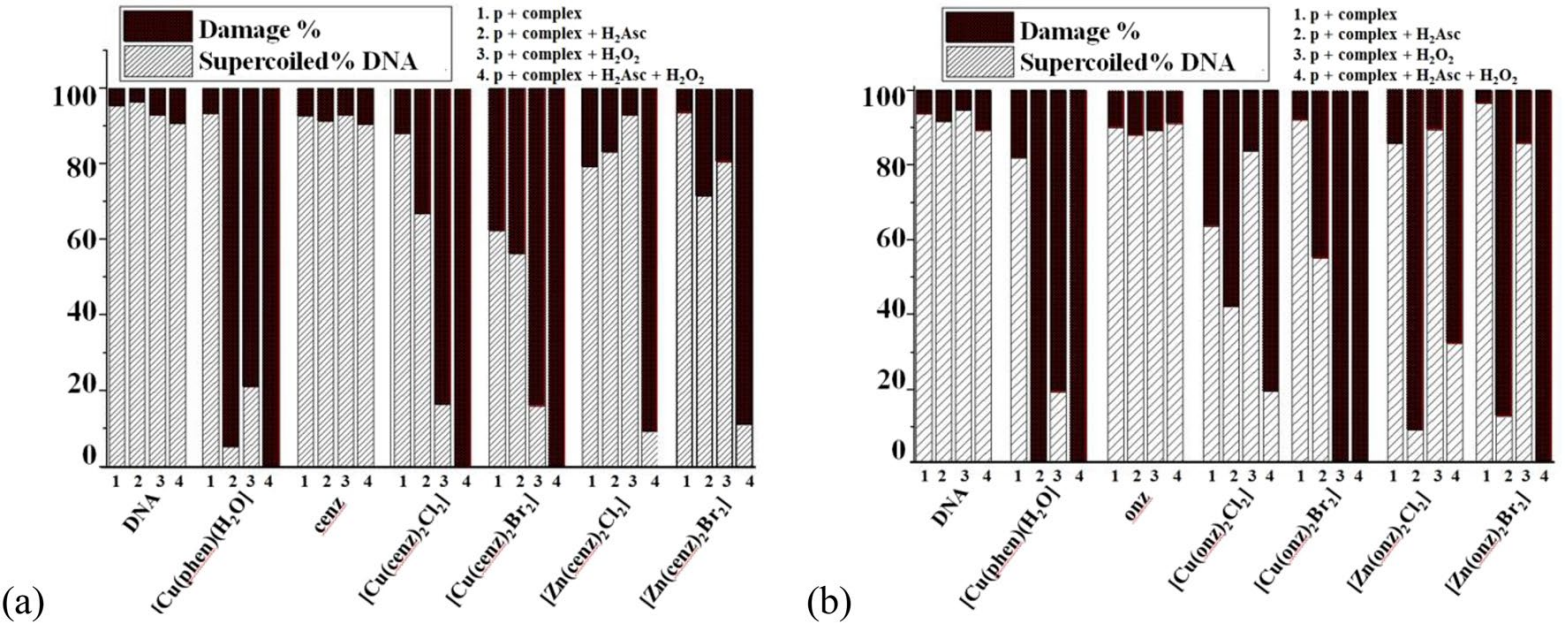
Percentages of supercoiled and damage DNA in the presence of the reference copper(II) complex, the free ligands cenz and onz, and the corresponding copper(II) and zinc(II) coordination compounds, at a concentration of 10 µM

The pure plasmid DNA presents ca. 90% of supercoiled form (I) and ca. 10% of circular nicked form (II). The controls corresponding to the DNA with H_2_Asc and peroxide show similar intensities (to those of the control), indicating that they do not produce DNA-damage (Fig. 11, lines 1, 2; Fig. 12, lines 1–3). [Cu(phen)_2_(H_2_O)](NO_3_)_2_ at a concentration of 10 μM was used as reference. The free ligands onz and cenz did not provoke any significant DNA cleavage (Fig. 11, lines 5, 6; Fig. 12, lines 6, 7). The electrophoresis assay shows that the amount of supercoiled DNA decreases in presence of the coordination compounds and H_2_Asc. In the case of the copper(II) compounds (Fig. 11 lines 8, 10, 12, 14), at concentration of 50 μM, the plasmid DNA is completely degraded into undetectable pieces (Fig. 11 lines 10, 14). The zinc(II) complexes exhibited a similar behavior (Fig. 11, lines 16, 18, 20, 22). When H_2_Asc + H_2_O_2_ are added, a significant DNA cleavage is observed. At a concentration of 10 μM, the plasmid is completely degraded, for the two series of complexes (Tables S2 and S4).

DNA degradation observed with the non-redox zinc(II) complex may be explained by the reduction of the nitro group to the nitro radical anion R-NO_2_^−^⋅ in the presence of H_2_Asc, which is most likely facilitated by the coordination of cenz to Zn(II) ion. Additionally, the addition of H_2_O_2_ resulted in a decrease of the supercoiled form, suggesting a higher DNA-damage, probably due to the formation of OH^−^⋅ species (Fig. 12). The copper(II) coordination compounds presented a higher activity, which may be expected as these complexes are redox-active and can therefore reduce ROS through a Fenton-like reaction. The data for all the investigated compounds are listed in Tables S-S4.

## Concluding remarks

Tetrahedral coordination compounds with copper(II) and zinc(II) were synthesized with the ligands cenz and onz. The complexes are stable in solution, with positive LogD_7.4_ values, which are in the appropriate range for a passive diffusion through the cell membrane.

[Cu(onz)_2_Cl_2_] and [Cu(onz)_2_Br_2_], exhibit very good activity against *toxoplasma gondii* in its tachyzoite morphology. Additionally, the in vitro studies against healthy host cell and in vivo acute toxicity assays showed low toxicity for these compounds. The preliminary promising results achieved certainly deserve additional in-depth biological studies which will be carried out in the near future.

DNA-binding studies reveal that the copper(II) compounds strongly interact with the minor groove of the biomolecule, while the interaction of weaker with the zinc(II) compounds which bind trough electrostatic contacts. These different type of binding are corroborated by the DNA-damage observed by gel electrophoresis, which showed that the zinc(II) complexes are less active than the copper(II) ones. Moreover, it was found that the coordination of the metal ion to the ligands seems to favor the reduction of the nitro group to NO_2_^−^⋅, and the generation of OH^−^⋅ species in the case of copper(II) in the presence of H_2_O_2_ and ascorbic acid, through a Fenton-like reaction (copper(II) to copper(I) redox cycle). Further studies are definitively required to assess the mechanism of formation of these radical species.

### Supplementary Information

Below is the link to the electronic supplementary material.

Supplementary file1 (PDF 947 KB)

## Data Availability

The crystallographic data for the structures have been deposited at the Cambridge Crystallographic Data Centre as supplementary publication CCDC 2293450 and 2272007. Copies of the data can be obtained free of charge via www.ccdc.cam.ac.uk/data_request/cif. The data that support the findings of this study are available from the corresponding author upon reasonable request.
