# Supplementary material for: Anti-toxoplasma activity and DNA-binding of copper(II) and zinc(II) coordination compounds with 5-nitroimidazole-based ligands
Source: J Biol Inorg Chem. 2023 Dec 15;29(1):33–49. doi: 10.1007/s00775-023-02029-7 (PMC11001709; doi:10.1007/s00775-023-02029-7)
Supplement: Supplementary file 1 — Supplementary file1 (PDF 947 KB) [file 775_2023_2029_MOESM1_ESM.pdf]

Anti-toxoplasma activity and DNA-binding of copper(II) and zinc(II) coordination compounds with 5-nitroimidazole-based ligands

Supplementary Information

Rubí Navarro-Peñaloza[1], Jhony Anacleto-Santos[2], Norma Rivera-Fernández[2], Francisco Sánchez-Bartez[3], Isabel Gracia-Mora[3], Ana B. Caballero[4], Patrick Gamez[4,5,6], Norah Barba-Behrens[1*]

[1]Departamento de Química Inorgánica, Facultad de Química, Universidad Nacional Autónoma de México, Ciudad Universitaria, Coyoacán, 04510, Ciudad de México, México.

[2]Departamento de Microbiología y Parasitología, Facultad de Medicina, Universidad Nacional Autónoma de México, Ciudad Universitaria, Coyoacán, 04510 Ciudad de México, México.

[3]Unidad de Investigación Preclínica (UNIPREC), Facultad de Química, Universidad Nacional Autónoma de México, Ciudad Universitaria, Coyoacán, 04510, Ciudad de México, México.

[4]nanoBIC, Departament de Química Inorgànica i Orgànica, Secció Química Inorgànica, Universitat de Barcelona, Martí i Franquès 1−11, 08028, Barcelona, Spain.

[5]Institute of Nanoscience and Nanotechnology (IN2UB), Universitat de Barcelona, 08028, Barcelona, Spain.

[6]Catalan Institution for Research and Advanced Studies (ICREA), Passeig Lluís Companys 23, 08010 Barcelona, Spain.

*Corresponding author. Norah Barba-Behrens, norah@unam.mx

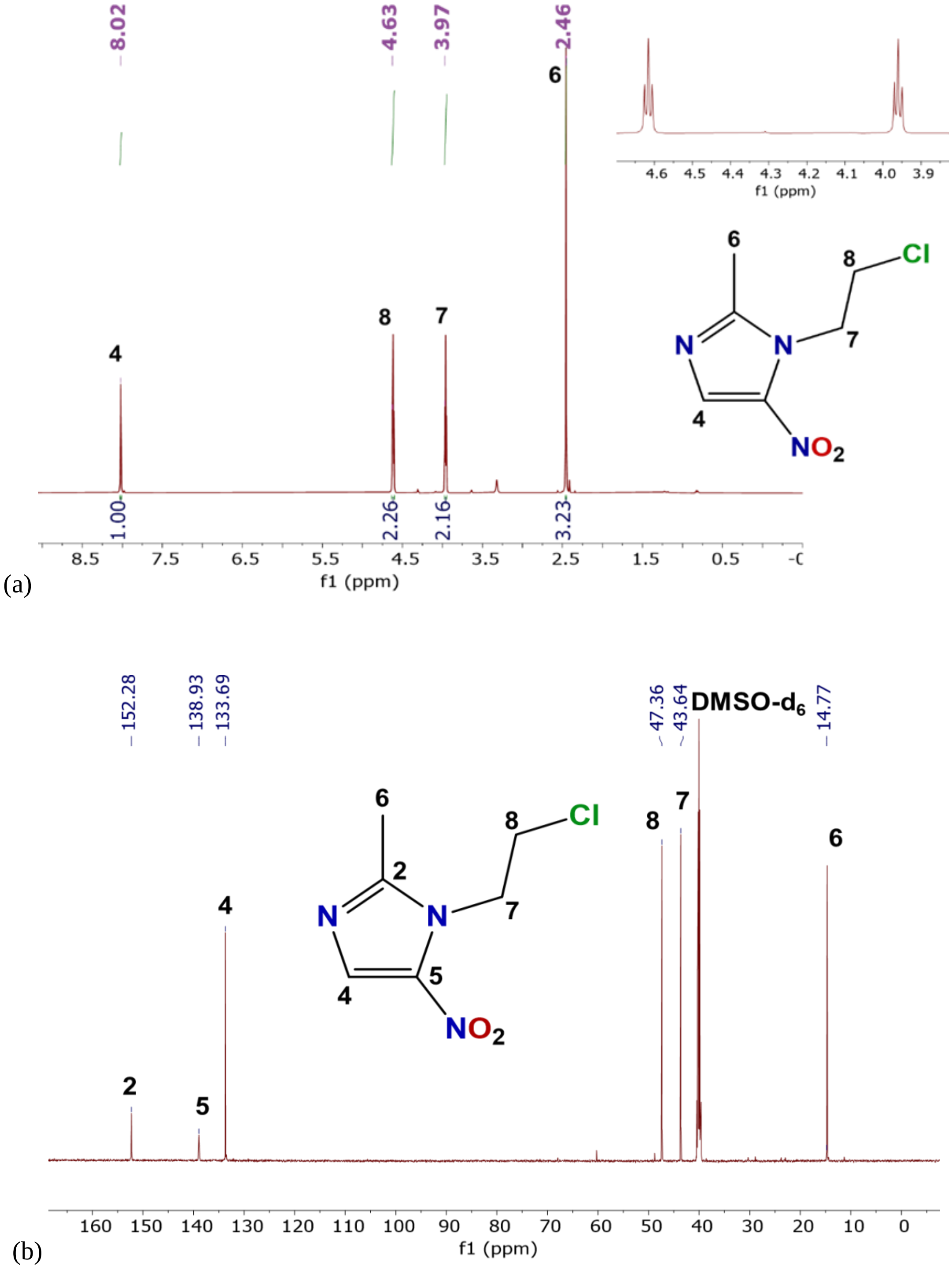

Fig. S1. (a) ^{1}H NMR and (b) ^{13}C NMR spectra of cenz ligand, d^{6}-DMSO, 400 MHz.

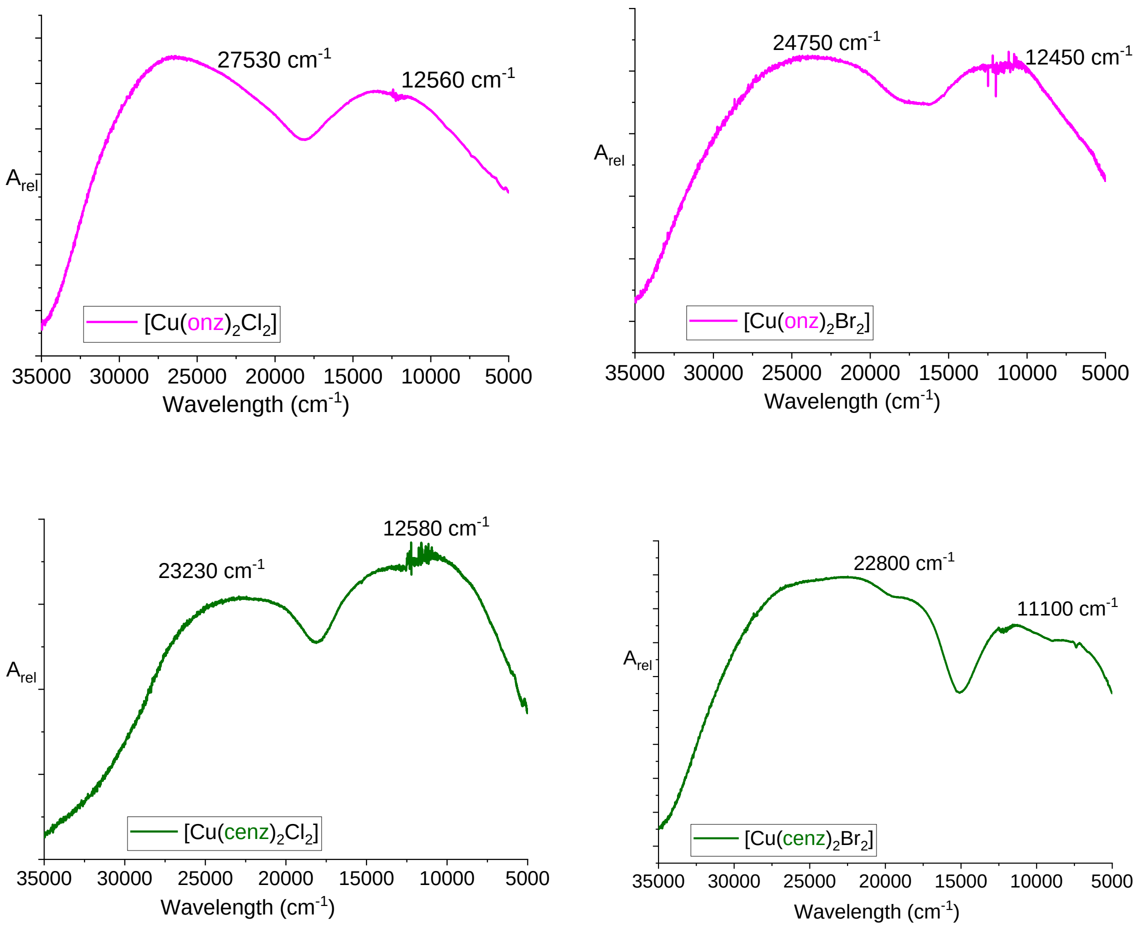

Fig. S2. Diffuse reflectance UV-Vis-NIR spectra for copper(II) coordination compounds.

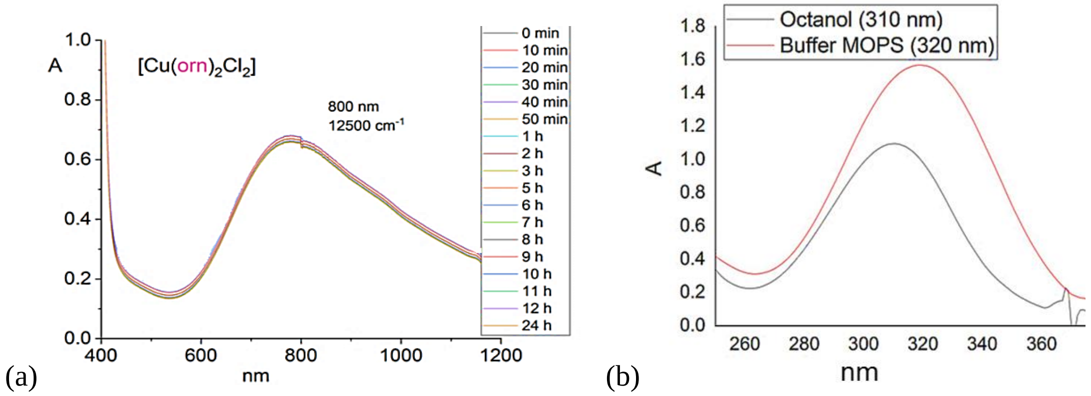

Fig. S3. (a) Electronic spectra of solution stability assays for [Cu(onz)$_2$Cl$_2$] (24 h), (b) Charge transfer bands for [Zn(onz)$_2$Cl$_2$] in octanol and MOPS buffer.

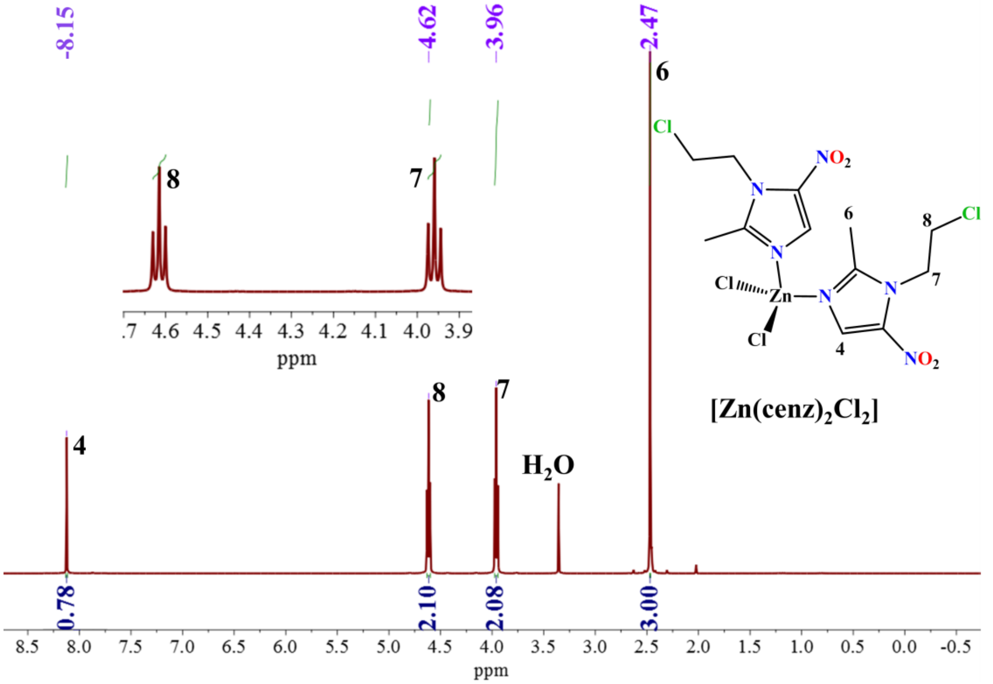

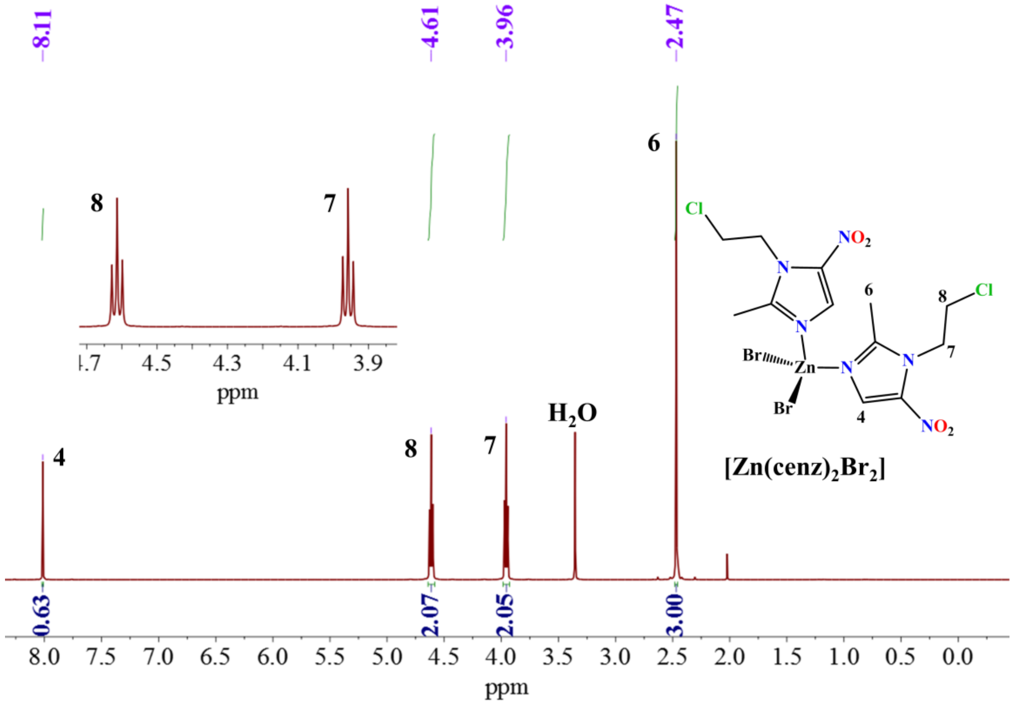

Fig. S4. ^{1}H NMR spectra for the zinc(II) coordination compounds with cenz, d^{6}-DMSO at 400 MHz.

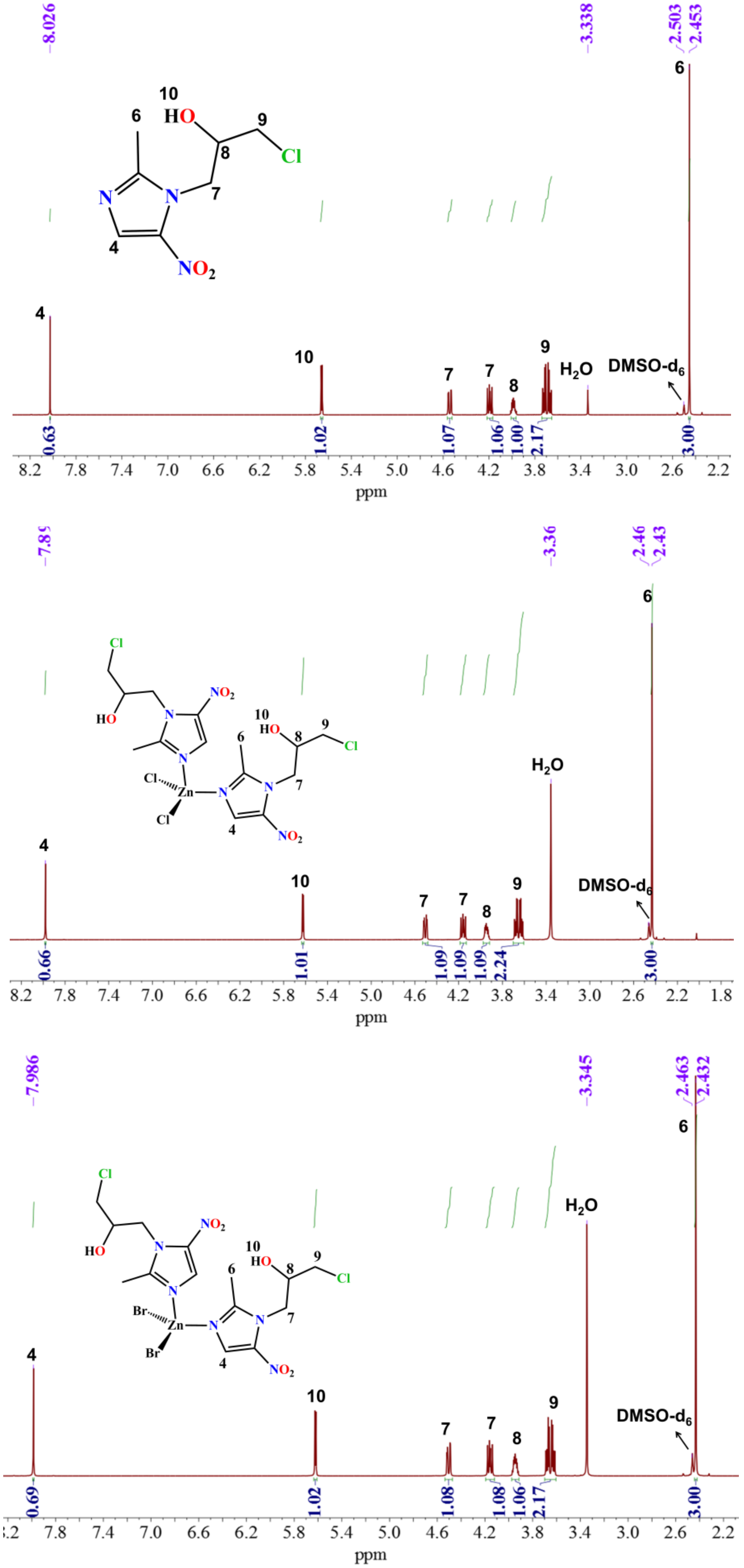

Fig. S5. ^{1}H NMR spectra for the onz and the zinc(II) complexes, d^6-DMSO at 400 MHz.

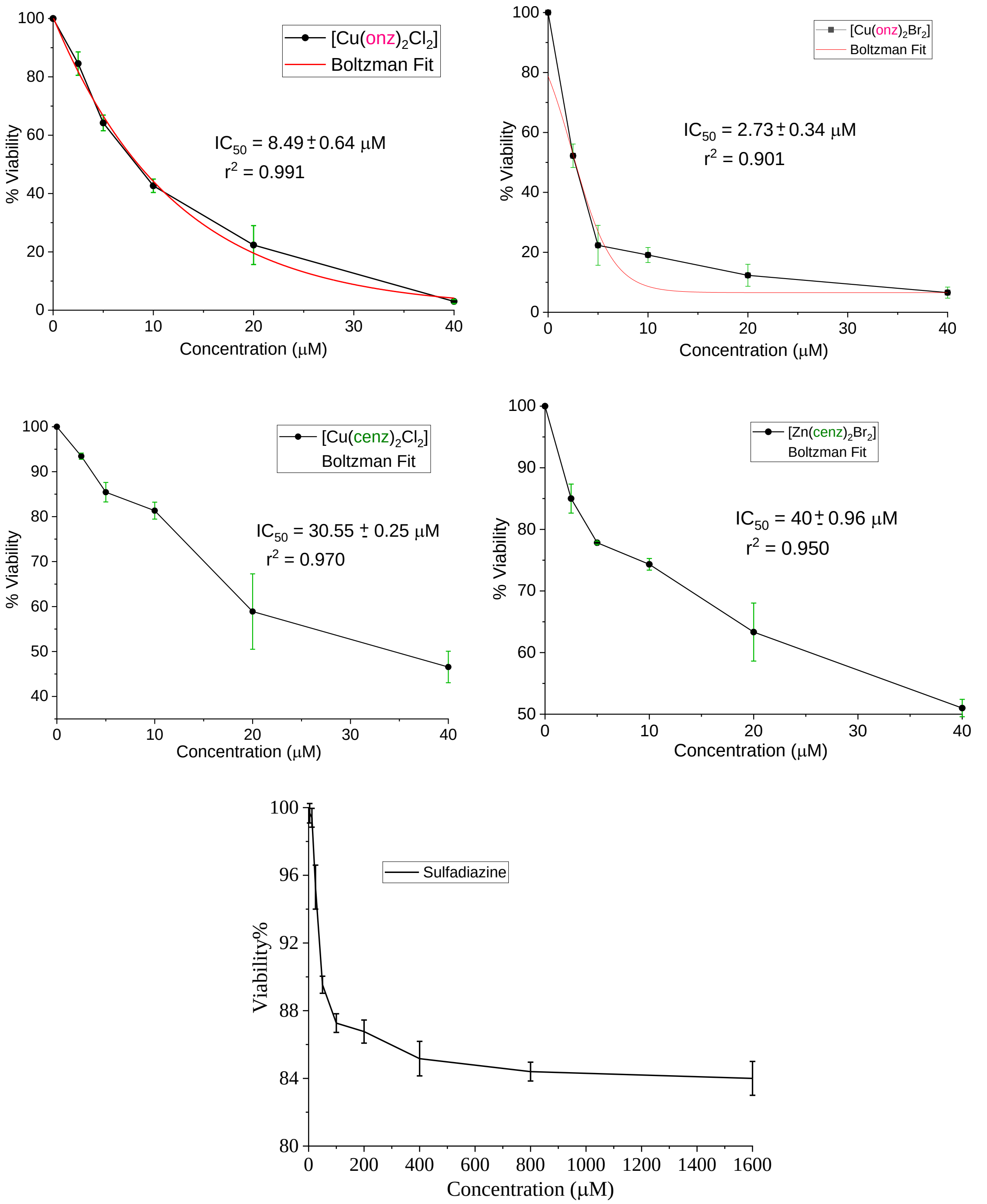

Fig. S6. Boltzmann regression analysis for the active coordination compounds and viability of sulfadiazine against *Toxoplasma gondii*.

Table S1. Gel electrophoresis results for onz and their coordination compounds with 15 μM_{pb} of pBR322 plasmid after 1 h of incubation at 30 °C in cacodylate-NaCl buffer pH=7.2.

	Gel line	Concentration (mM)	%Supercoiled	%Nicked	%Damage
1.	plasmid DNA (p)	-	93.60	5.90	6.40
2.	p + H_2Asc	-	91.50	-	8.50
3.	Cu(phen)	-	81.70	-	18.30
4.	Cu(phen) + H_2Asc	-	-	-	100.00
5.	p + onz	50	90.20	7.60	9.80
6.	p + onz + H_2Asc	50	88.20	5.60	11.80
7.	p + $[Cu(onz)_2Cl_2]$	10	63.60	6.40	36.40
8.	p + $[Cu(onz)_2Cl_2]$+ H_2Asc	10	41.90	33.00	58.10
9.	p + $[Cu(onz)_2Cl_2]$	50	66.20	9.70	33.80
10.	p + $[Cu(onz)_2Cl_2]$ + H_2Asc	50	-	-	100.00
11.	p + $[Cu(onz)_2Br_2]$	10	92.20	-	7.80
12.	p + $[Cu(onz)_2Br_2]$+ H_2Asc	10	55.30	35.60	44.70
13.	p + $[Cu(onz)_2Br_2]$	50	93.30	23.00	6.70
14.	p + $[Cu(onz)_2Br_2]$+ H_2Asc	50	-	-	100.00
15.	p + $[Zn(onz)_2Cl_2]$	10	85.40	-	14.6
16.	p + $[Zn(onz)_2Cl_2]$ + H_2Asc	10	8.80	65.70	91.2
17.	p + $[Zn(onz)_2Cl_2]$	50	67.10	3.90	32.9
18.	p + $[Zn(onz)_2Cl_2]$ + H_2Asc	50	-	4.80	100
19.	p + $[Zn(onz)_2Br_2]$	10	96.50	2.60	3.5
20.	p + $[Zn(onz)_2Br_2]$ + H_2Asc	10	12.70	81.80	87.3
21.	p + $[Zn(onz)_2Br_2]$	50	88.10	0.40	11.9
22.	p + $[Zn(onz)_2Br_2]$ + H_2Asc	50	1.80	98.80	98.2

Table S2. Gel electrophoresis results for onz and their coordination compounds with 15 μM_{pb} of pBR322 plasmid after 1 h of incubation at 30 °C in cacodylate-NaCl buffer pH=7.2 with H_2O_2.

	Gel line	Concentration (mM)	%Supercoiled	%Nicked	%Damage
1.	plasmid DNA (p)	-	90.10	4.40	9.90
2.	p + H_2O_2	-	94.60	4.50	5.40
3.	p + H_2Asc + H_2O_2	-	89.20	9.00	10.80
4.	Cu(phen) + H_2O_2	-	19.30	17.20	80.70
5.	Cu(phen) + H_2Asc + H_2O_2		-	-	100.00
6.	p + onz + H_2O_2	50	89.30	8.90	10.70
7.	p + onz + H_2Asc + H_2O_2	50	91.20	4.30	8.80
8.	p + [Cu(onz)$_2$Cl$_2$] + H_2O_2	10	83.60	6.10	16.40
9.	p + [Cu(onz)$_2$Cl$_2$]+ H_2Asc + H_2O_2	10	19.40	46.50	80.60
10.	p + [Cu(onz)$_2$Cl$_2$] + H_2O_2	50	-	-	100.00
11.	p + [Cu(onz)$_2$Cl$_2$] + H_2Asc + H_2O_2	50	-	-	100.00
12.	p + [Cu(onz)$_2$Br$_2$] + H_2O_2	10	-	-	100.00
13.	p + [Cu(onz)$_2$Br$_2$]+ H_2Asc + H_2O_2	10	-	-	100.00
14.	p + [Cu(onz)$_2$Br$_2$] + H_2O_2	50	-	-	100.00
15.	p + [Cu(onz)$_2$Br$_2$]+ H_2Asc	50	-	-	100.00
16.	p + [Zn(onz)$_2$Cl$_2$] + H_2O_2	10	89.20	5.90	10.80
17.	p + [Zn(onz)$_2$Cl$_2$] + H_2Asc + H_2O_2	10	32.10	44.00	67.90
18.	p + [Zn(onz)$_2$Cl$_2$] + H_2O_2	50	21.70	47.60	78.30
19.	p + [Zn(onz)$_2$Cl$_2$] + H_2Asc + H_2O_2	50	-	-	100.00
20.	p + [Zn(onz)$_2$Br$_2$] + H_2O_2	10	85.70	4.70	14.30
21.	p + [Zn(onz)$_2$Br$_2$] + H_2Asc + H_2O_2	10	-	-	100.00
22.	p + [Zn(onz)$_2$Br$_2$] + H_2O_2	50	55.20	29.10	44.80
23.	p + [Zn(onz)$_2$Br$_2$] + H_2Asc + H_2O_2	50	-	-	100.00

Table S3. Gel electrophoresis results for cenz and their coordination compounds with 15 μM_{pb} of pBR322 plasmid after 1 h of incubation at 30 °C in cacodylate-NaCl buffer pH=7.2.

Gel line		Concentration (mM)	%Supercoiled	%Nicked	%Damage
1.	plasmid DNA (p)	-	95.20	-	4.80
2.	p + H_2Asc	-	96.30	-	3.70
3.	Cu(phen)	-	93.20	-	6.80
4.	Cu(phen) + H_2Asc	-	5.10	64.80	94.90
5.	p + cenz	50	92.50	-	7.50
6.	p + cenz + H_2Asc	50	91.20	-	8.80
7.	p + [Cu(cenz)$_2$Cl$_2$]	10	88.00	-	12.00
8.	p + [Cu(cenz)$_2$Cl$_2$]+ H_2Asc	10	66.80	16.60	33.20
9.	p + [Cu(cenz)$_2$Cl$_2$]	50	68.50	-	31.50
10.	p + [Cu(cenz)$_2$Cl$_2$] + H_2Asc	50	-	-	100.00
11.	p + [Cu(cenz)$_2$Br$_2$]	10	62.20	11.30	37.80
12.	p + [Cu(cenz)$_2$Br$_2$]+ H_2Asc	10	56.10	-	43.90
13.	p + [Cu(cenz)$_2$Br$_2$]	50	66.20	7.50	33.80
14.	p + [Cu(cenz)$_2$Br$_2$]+ H_2Asc	50	-	-	100.00
15.	p + [Zn(cenz)$_2$Cl$_2$]	10	79.20	2.50	20.80
16.	p + [Zn(cenz)$_2$Cl$_2$] + H_2Asc	10	82.90	2.90	17.10
17.	p + [Zn(cenz)$_2$Cl$_2$]	50	81.60	2.20	18.40
18.	p + [Zn(cenz)$_2$Cl$_2$] + H_2Asc	50	19.40	40.60	80.60
19.	p + [Zn(cenz)$_2$Br$_2$]	10	93.80	3.00	6.20
20.	p + [Zn(cenz)$_2$Br$_2$] + H_2Asc	10	71.60	6.40	28.40
21.	p + [Zn(cenz)$_2$Br$_2$]	50	88.40	2.20	11.60
22.	p + [Zn(cenz)$_2$Br$_2$] + H_2Asc	50	3.90	39.50	96.10

Table S4. Gel electrophoresis results for cenz and their coordination compounds with 15 μM_{pb} of pBR322 plasmid after 1 h of incubation at 30 °C in cacodylate-NaCl buffer pH=7.2 with H_2O_2.

	Gel line	Concentration (mM)	%Supercoiled	%Nicked	%Damage
1.	plasmid DNA (p)	-	90.00	9.30	10.00
2.	p + H_2O_2		92.70	6.50	7.30
3.	p + H_2Asc + H_2O_2	-	90.50	3.90	9.50
4.	Cu(phen) + H_2O_2	-	21.00	64.80	79.00
5.	Cu(phen) + H_2Asc + H_2O_2	-	-	-	100.00
6.	p + cenz + H_2O_2	50	92.80	5.90	7.20
7.	p + cenz + H_2Asc + H_2O_2	50	90.30	4.00	9.70
8.	p + [Cu(cenz)$_2$Cl$_2$] + H_2O_2	10	16.50	84.30	83.50
9.	p + [Cu(cenz)$_2$Cl$_2$]+ H_2Asc + H_2O_2	10	-	-	100.00
10.	p + [Cu(cenz)$_2$Cl$_2$] + H_2O_2	50	-	-	100.00
11.	p + [Cu(cenz)$_2$Cl$_2$] + H_2Asc + H_2O_2	50	-	-	100.00
12.	p + [Cu(cenz)$_2$Br$_2$] + H_2O_2	10	16.00	37.10	84.00
13.	p + [Cu(cenz)$_2$Br$_2$]+ H_2Asc + H_2O_2	10	-	-	100.00
14.	p + [Cu(cenz)$_2$Br$_2$] + H_2O_2	50	-	-	100.00
15.	p + [Cu(cenz)$_2$Br$_2$]+ H_2Asc + H_2O_2	50	-	-	100.00
16.	p + [Zn(cenz)$_2$Cl$_2$] + H_2O_2	10	92.70	6.50	7.30
17.	p + [Zn(cenz)$_2$Cl$_2$] + H_2Asc + H_2O_2	10	9.10	2.10	90.90
18.	p + [Zn(cenz)$_2$Cl$_2$] + H_2O_2	50	2.40	81.50	97.60
19.	p + [Zn(cenz)$_2$Cl$_2$] + H_2Asc + H_2O_2	50	-	-	100.00
20.	p + [Zn(cenz)$_2$Br$_2$] + H_2O_2	10	80.70	27.20	19.30
21.	p + [Zn(cenz)$_2$Br$_2$] + H_2Asc + H_2O_2	10	11.00	75.80	89.00
22.	p + [Zn(cenz)$_2$Br$_2$] + H_2O_2	50	1.60	63.30	98.40
23.	p + [Zn(cenz)$_2$Br$_2$] + H_2Asc + H_2O_2	50	21.60	10.40	78.40
